# Proficiency testing and cross-laboratory method comparison to support standardisation of diatom DNA metabarcoding for freshwater biomonitoring

**DOI:** 10.3897/mbmg.9.133264

**Published:** 2025-01-10

**Authors:** Valentin Vasselon, Sinziana F. Rivera, Éva Ács, Salomé Baja Almeida, Karl B. Andree, Laure Apothéloz-Perret-Gentil, Bonnie Bailet, Ana Baričević, Kevin K. Beentjes, Juliane Bettig, Agnès Bouchez, Camilla Capelli, Cécile Chardon, Mónika Duleba, Tina Elersek, Clémence Genthon, Maša Jablonska, Louis Jacas, Maria Kahlert, Martyn G. Kelly, Jan-Niklas Macher, Federica Mauri, Marina Moletta-Denat, Andreia Mortágua, Jan Pawlowski, Javier Pérez-Burillo, Martin Pfannkuchen, Erik Pilgrim, Panayiota Pissaridou, Frédéric Rimet, Karmen Stanic, Kálmán Tapolczai, Susanna Theroux, Rosa Trobajo, Berry Van der Hoorn, Marlen I. Vasquez, Marie Vidal, David Wanless, Jonathan Warren, Jonas Zimmermann, Benoît Paix

**Affiliations:** 1 Scimabio-Interface, Thonon-Les-Bains, France; 2 Swiss Federal Research Institute WSL Agroscope, Birmensdorf, Switzerland; 3 Faculty of Water Sciences, University of Public Service, Baja, Hungary; 4 GeoBioTec and Biology Department, University of Aveiro, Aveiro, Portugal; 5 IRTA, La Ràpita, Spain; 6 Agroscope, Nyon, Switzerland; 7 UMR DECOD, INRAE, Rennes, France; 8 Ruder Boskovic Institute, Center for Marine Research, Laboratory for Evolutionary Ecology (LEE), Rovinj, Croatia; 9 Naturalis Biodiversity Center, Leiden, Netherlands; 10 Botanic Garden and Botanical Museum, Freie Universität Berlin, Berlin, Germany; 11 UMR CARRTEL, INRAE, Université de Savoie Mont-Blanc, Thonon-Les-Bains, France; 12 SUPSI, Institute of Earth Sciences, Mendrisio, Switzerland; 13 National Institute of Biology, Ljubljana, Slovenia; 14 INRAE Transfert - Metys- Service Génomique Plateforme GeT-PlaGe, Castanet-Tolosan, France; 15 University of Ljubljana, Ljubljana, Slovenia; 16 Department Aquatic Sciences and Assessment, Swedish University of Agricultural Sciences, Uppsala, Sweden; 17 Bowburn Consultancy, University of Nottingham, Durham, UK; 18 SUPSI, Institute of Microbiology, Mendrisio, Switzerland; 19 INRAE Transfert - Metys- LBE- Service Génomique, Narbonne, France; 20 Department of Genetics and Evolution, University of Geneva, Geneva, Switzerland; 21 US Environmental Protection Agency, Cincinnati, USA; 22 Cyprus University of Technology, Limassol, Cyprus; 23 HUN-REN Balaton Limnological Research Institute, Tihany, Hungary; 24 SCCWRP, Costa Mesa, USA; 25 Environment Agency, Bristol, UK

**Keywords:** Cross-laboratory experiment, DNA-based approach, ecological status assessment, intercalibration, standardisation

## Abstract

DNA metabarcoding of benthic diatoms has been successfully applied for biomonitoring at the national scale and can now be considered technically ready for routine application. However, protocols and methods still vary between and within countries, limiting their transferability and the comparability of results. In order to overcome this, routine use of DNA metabarcoding for diatom biomonitoring requires knowledge of the sources of variability introduced by the different steps of the procedure. Here, we examine how elements of routine procedures contribute to variability between European laboratories. A set of four experiments were performed focusing on DNA extraction and PCR amplification steps to evaluate their reproducibility between different laboratories and the variability introduced by different protocols currently applied by the scientific community. Under the guidance of a reference laboratory, 17 participants from 14 countries performed DNA extraction and PCR amplification in parallel, using the same fixed protocol and their own choice of protocol. Experiments were performed by each participant on a set of standardised DNA and biofilm samples (river, lake and mock community) to investigate potential systematic and random errors. Our results revealed the successful transferability of a protocol amongst labs and a highly similar and consistent ecological assessment outcome obtained regardless of the protocols used by each participant. We propose an “all for one but prove them all” strategy, suggesting that distinct protocols can be used within the scientific community, as long as their consistency is be proven by following minimum standard requirements.

## Introduction

Genetic approaches have recently emerged for monitoring biodiversity in aquatic ecosystems (Baird and Hajibabaei 2012; Thomsen et al. 2012; Carew et al. 2013; Mächler et al. 2014; Valentini et al. 2016 Taberlet et al. 2018; Meng et al. 2019; Miya 2022; Kelly et al. 2024). These methods provide genetic and taxonomic information about biological communities by extracting DNA from environmental samples such as water, biofilms and soil (Taberlet et al. 2018). In the context of the European Water Framework Directive (WFD, 2000/60/EC), genetic methods are now employed to assess the ecological health of aquatic ecosystems by monitoring Biological Quality Elements (BQE) including phytoplankton, macrophytes and phytobenthos, benthic invertebrate fauna and fish (Hering et al. 2018; Vasselon et al. 2019; Kelly et al. 2020a; Pont et al. 2021; Duarte et al. 2023). These elements are integral to national monitoring programmes within the extensive European network, encompassing 110,000 surface water monitoring sites, including 79.5% rivers and 11% lake sites (Charles et al. 2021). High-throughput methods have the potential to enhance spatio-temporal monitoring and expedite information transfer to water managers, contributing to increased protection of aquatic ecosystems (Carraro et al. 2020, 2023; Seymour et al. 2021). Amongst the BQEs, benthic diatoms as part of phytobenthos BQE are widely employed for freshwater quality assessment due to their sensitivity to a variety of ecological conditions (Pawlowski et al. 2018; Rivera et al. 2020).

Significant progress has been made in developing DNA metabarcoding techniques for assessing benthic diatom assemblages. Technological advancements and optimised protocols have allowed the acquisition of robust taxonomic (species) and genetic (Operational Taxonomic Unit - OTU, Amplicon Sequence Variant - ASV) information, from which diatom quality indices can be calculated in order to infer ecological status of rivers and lakes (Apothéloz-Perret-Gentil et al. 2017; Keck et al. 2018; Tapolczai et al. 2019; Feio et al. 2020). Despite the difficulties to complete diatom barcode reference library, due to the important species diversity in the environment estimated to more than 100,000 species (Mann and Vanormelingen 2013), while only close around 18,000 species are described (Guiry 2024), strategies were proposed to accelerate the barcode completion of abundant taxa that are the main contributors to diatom indices (Rimet et al. 2018; Weigand et al. 2019). Diatom DNA metabarcoding has been successfully tested within national river monitoring networks in Europe, indicating its principle readiness for routine application, providing the most abundant taxa are represented in the DNA sequence database (e.g. Apothéloz-Perret-Gentil et al. (2017); Bailet et al. (2019); Mortágua et al. (2019); Vasselon et al. (2019); Kelly et al. (2020a); Pérez-Burillo et al. (2020); Rivera et al. (2020); Pissaridou et al. (2021)). However, variations in protocols and methods between different laboratories, both within and between countries, may hinder method transferability and result comparability. Therefore, standardisation of all processing steps, from sample collection to the final ecological status assessment, is crucial for routine DNA metabarcoding in diatom monitoring. Standardisation should focus on the minimum requirements that are essential to obtain reliable data. Several studies have already investigated the variability introduced by certain key steps in community structure analysis using DNA metabarcoding. These steps include sample preservation (Baricevic et al. 2022), DNA extraction method (Vasselon et al. 2017), genetic barcode comparison (Kermarrec et al. 2013; Pérez-Burillo et al. 2022) and bioinformatics (Tapolczai et al. 2019; Bailet et al. 2020; Rivera et al. 2020). However, certain crucial steps, such as PCR amplification, have not yet been investigated. Therefore, it is important to gather data on and understand the differences caused by the variability introduced by these steps in order to propose guidelines for future standardisation, particularly for regulatory monitoring purposes (Blackman et al. 2019, 2024; Thalinger et al. 2021; Bunholi et al. 2023).

Intercalibration exercises and proficiency tests have been long used to harmonise knowledge and results within the scope of the WFD. While these exercises are standard practice for morphological intercomparison (e.g. European Commission (2008, 2013); Kelly et al. (2009); Kahlert et al. (2012); Almeida et al. (2014)), there is a lack of such exercises conducted using DNA-based approaches for specific applications in the WFD context. Proficiency tests, organised as cross-laboratory comparisons, are essential for assessing participants’ experiment performance, adhering to established normative guidelines (e.g. ISO 13528 2015, 2022).

Building on previous initiatives that led to the development of a CEN technical report for the routine sampling of benthic diatoms from rivers and lakes adapted for metabarcoding analyses (CEN/TR 17245 2018), a series of experiments was launched during the EU-funded COST Action DNAqua-Net (Leese et al. 2016) WG2 diatom workshop in Cyprus (organized in 2019). Briefly, the aim of DNAqua-Net was to build a community of researchers and strengthen their interactions to develop the application of DNA-based monitoring approaches and future guidelines for its standardisation. Our experiments focused on DNA extraction and amplification procedures as they are the first of the laboratory stages of DNA metabarcoding where there is known to be a wide diversity of methods that might introduce variability into results (Vasselon et al. 2017; Nagai et al. 2022). The two main objectives were to: (i) assess the reproducibility of a standardised protocol across various laboratories (“intercalibration as proficiency test”) and (ii) evaluate the variability introduced by the numerous protocols currently utilised for diatom DNA metabarcoding for ecological assessment (“method intercomparison”).

To this end, 17 laboratories from 14 countries performed DNA extraction and PCR amplification simultaneously, under the guidance of a reference laboratory agreed by the participants (INRAE CARRTEL, France). Participants followed stringent quality control measures and adhered to established standards and protocols. Each utilised both an agreed standard lab protocol and their own choice of protocol. Each participant conducted experiments on a standardised set of DNA and biofilm samples, encompassing samples from a river, a lake and a mock community.

In order to assess the variability in DNA extraction and PCR amplification steps, all other metabarcoding process steps were standardised and MiSeq sequencing preparation was performed by the reference laboratory. Variability within and between participants was assessed in terms of DNA extract quantity, taxonomic (genus, species) and genetic richness, as well as community structure comparison and diatom quality index scores (IPS and IBD). Additionally, the impact of the different DNA extraction and PCR amplification protocols on the diatom quality index scores and the final ecological status assessment was examined.

The objective of this collaborative effort extended beyond assessing individual participant performance. We aimed to determine whether a molecular method could be readily transferred between different laboratories for potential operational use in routine freshwater monitoring. While the results do not aim to prescribe a universally applicable protocol, they provide valuable insights for establishing guidelines and minimum requirements for conducting diatom metabarcoding for biomonitoring. The primary focus of the cross-laboratory experiments is to evaluate method variability and transferability in a controlled environment, rather than favouring any specific laboratory or molecular technique.

## Material and methods

### Organisation of the cross-laboratory experiments

Organisation of a collaborative international cross-laboratory experiment focusing on diatom DNA metabarcoding for biomonitoring was agreed during the COST DNAqua-Net WG2 workshop held in CUT, Limassol (Cyprus) in 2019. A core organising team, composed of CUT, OFB and Scimabio Interface, along with the reference laboratory (RL, affiliation: INRAE CARRTEL), was designated to lead and coordinate the experiments. Seventeen laboratories from 14 countries (referred to as A to Q) within the international DNAqua-Net community expressed interest in participating in the experiments. These laboratories represented both public and private institutes, with varying levels of expertise in the application of molecular methods. Additionally, the laboratories had diverse experience in applications in research and operational freshwater monitoring. Major service providers were also invited to contribute to the experiments (e.g. Macherey-Nagel, Takara).

**Figure 1.**
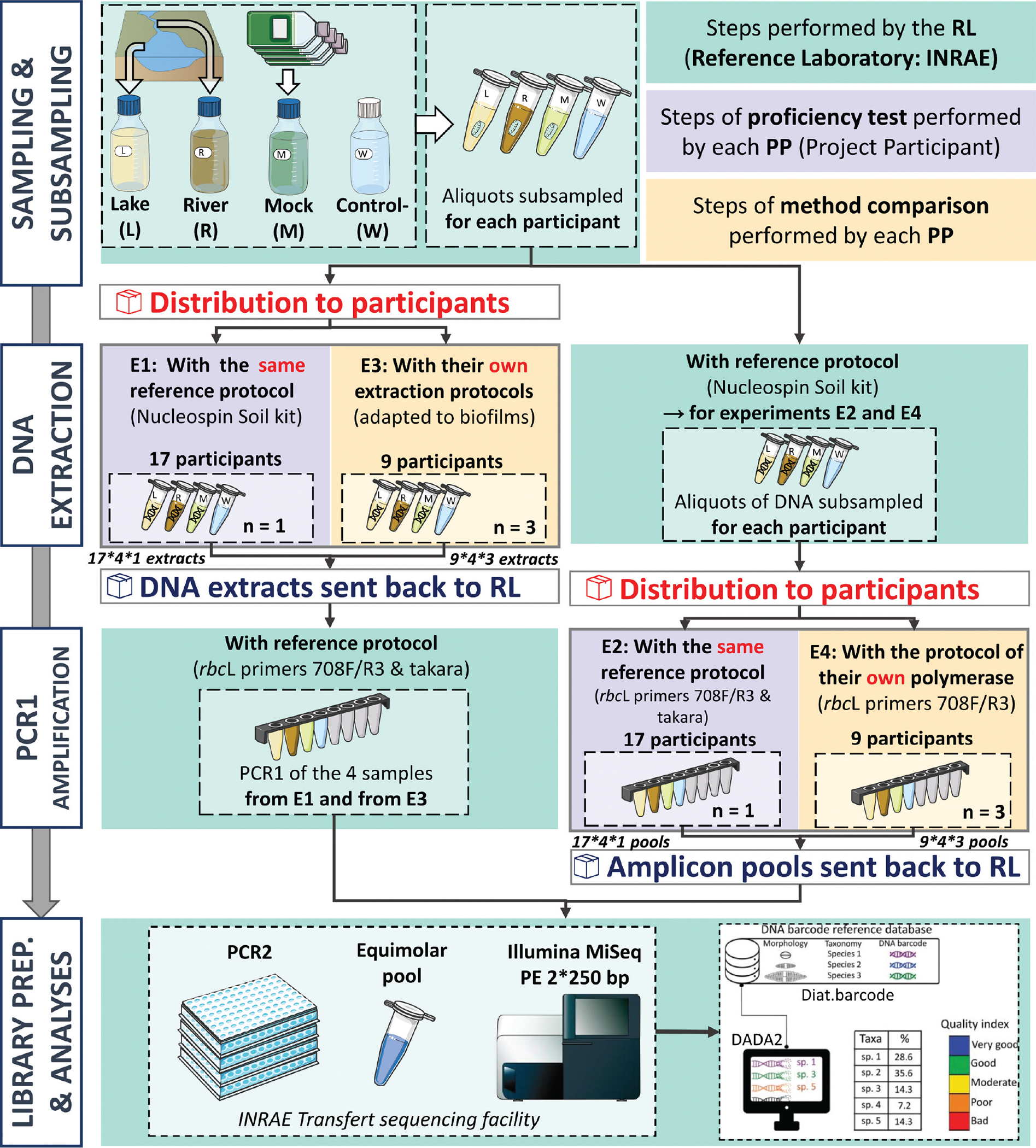
Workflow of the experimental design of the study (experiments E1-E4). This details the steps of the proficiency test (blue), the method comparison (orange) and those related to the organisation of the whole experiment by the reference lab (RL, green). L, R, M and W labels correspond to the lake sample, river sample, mock community and negative controls, respectively.

Two categories of international cross-laboratory experiments were conducted: proficiency tests and method comparison. The “proficiency tests” experiments were designed to assess the performance of each participant using the same molecular protocols (DNA extraction and PCR amplification), thus providing information regarding the transferability and the reproducibility of the tested protocol. The “method comparison” experiments were designed to evaluate the variability introduced when using different molecular protocols (DNA extraction, PCR amplification), thus providing insights into the flexibility of molecular approaches. The organisation of cross-laboratory experiments involved several key phases (Fig. 1):

Phase 1: The core organising team designed the experiments and circulated laboratory protocols (DNA extraction, PCR amplification) and reagents (DNA extraction and Polymerase kits, metabarcoding primers, batch control) to all participating laboratories, along with calibrated samples preparation (biofilm, DNA extracts, see details in section Collection and preparation of samples). Each participant communicated their interest in contributing to the various experiments (E1 to E4, described below) to both the core team and the reference laboratory, which subsequently provided them with the corresponding experimental materials (Fig. 1).

Phase 2: After receiving the experimental materials, independent testing was carried out simultaneously by all participants and the reference laboratory. The experiments were conducted in parallel within a one-month period to minimise the potential effect of DNA and biofilm sample degradation.

Phase 3: All experimental outputs (data) and products (DNA extracts, PCR amplicons) were submitted by each participant to the reference laboratory, which finalised library preparation prior to high-throughput sequencing (HTS) and shipped them to the sequencing platforms.

Phase 4: After production of HTS data (fastq), bioinformatics treatments and downstream data analyses were performed by the reference laboratory.

### Collection and preparation of calibrated samples

To follow proficiency test statistical design (ISO 13528 2015, 2022) and ensure a maximum number of participants, experiments and replicates in the cross-laboratory test, a limited set of calibrated samples was prepared by the reference laboratory (Fig. 1). The samples included:

- Environmental biofilm samples (for experiments E1 and E3): Two biofilm samples were collected in March 2020 from Lake Geneva (“L” sample, 46.368054°N, 6.453779°E) and Edian River (“R” sample, 46.257849°N, 6.725084°E) following standard procedures (CEN/TR 17245 2018). To ensure an adequate volume of samples for all the experiments and participants, the number of stones collected per site was increased to 20, with a minimum surface area of 100 cm^2^ per stone scraped. The biofilm collected from stones was pooled into a 1 litre flask filled with 99% ethanol to achieve a final ethanol concentration of > 70% (CEN/TR 17245 2018; Baricevic et al. 2022). Subsequently, the R and L biofilm samples were homogenised on a magnetic stirrer for 5 minutes and then subsampled into 2 ml Eppendorf tubes. A total of 200 2 ml aliquots were prepared for each sample, which were stored in dark conditions at 4 °C during several weeks until they were used simultaneously by all the participants for the cross-laboratory DNA extraction experiments.
- Environmental DNA samples (for experiments E2 and E4): five units of R and seven units of L biofilm aliquots were individually extracted using the Nucleospin Soil kit (Macherey-Nagel) following the DNA extraction protocol available at https://dx.doi.org/10.17504/protocols.io.bd52i88e. Final elution was performed in 30 μl of SE solution and the sample replicates were pooled together to increase the volume of DNA extracts. Finally, L and R DNA extracts were subsampled into 10 μl aliquots and stored at −20 °C during several weeks prior to their use simultaneously by all the participants in the cross-laboratory experiments.
- Mock sample as biofilm (for experiments E1 and E3) and DNA positive controls (for experiments E2 and E4): A mock community with a known composition (“M” sample) was created by combining 12 pure diatom strains available in the Thonon Culture Collection (TCC https://carrtel-collection.hub.inrae.fr/ Suppl. material 1: table S1). Each diatom strain was cultivated in triplicate using 40 ml sterile DV medium in 50 ml Nunc^™^ EasYFlasks^™^ (Thermo Fisher Scientific), following the conditions described in Vasselon et al. (2018). Once the strains reached the plateau growth phase, the supernatant was removed from each vial to increase cell concentration. The replicates of each strain were pooled and filled with 95% pure ethanol to achieve a final ethanol concentration of 70% (CEN/TR 17245 2018; Baricevic et al. 2022). Then, the 12 ethanol-fixed strains were pooled together to create the M sample. Finally, the M sample was subsampled into 2 ml Eppendorf tubes and stored under dark conditions at 4 °C like the environmental biofilm samples. For DNA positive controls, a total of seven aliquots from the M sample were extracted and 10 μl DNA extracts were prepared from each aliquot following the same procedure as described previously for the L and R samples.
- Biofilm (for experiments E1 and E3) and DNA negative controls (for experiments E2 and E4) (DNA free water): free DNA molecular grade water was subsampled into 2 ml Eppendorf tubes and included as negative controls in the DNA extraction and PCR amplification experiments.

### Proficiency tests

We performed two experiments focused on the DNA extraction (E1) and PCR amplification (E2) steps to assess the consistency of the results obtained from different labs using the same samples and reagents. For brevity, we refer to these as proficiency tests. To simplify the experiments, the protocols used for DNA extraction from biofilm samples and PCR amplification of diatom communities corresponded to those routinely used by the reference laboratory for diatom metabarcoding and already applied by the scientific community (e.g. Bailet et al. (2019); Mortágua et al. (2019); Pérez-Burillo et al. (2020, 2022); Pissaridou et al. (2021)).

DNA extraction proficiency test experiment (E1): All participants applied the Nucleospin Soil kit (Macherey-Nagel, https://dx.doi.org/10.17504/protocols.io.bd52i88e) to extract DNA from four calibrated samples: river biofilm (R), lake biofilm (L), mock community (M) and DNA-free water (W) as the negative control (Table 1). A quantity of 1.8 ml of R and 1.9 ml of L, M and W samples were supplied to each participant in a 2 ml Eppendorf tube for extraction and each sample was extracted once. Importantly, the reference laboratory provided reagents (Nucleospin Soil kits) for DNA extraction from the same lot to each participant. After DNA extraction, each participant performed the final DNA elution into 30 μl of SE buffer. Then, participants assessed DNA quality and quantity using internal devices (data not shown) and 15 μl of each DNA extract was sent to the reference laboratory for downstream laboratory preparation. These instructions were given to the participants as described in Suppl. material 1: fig. S1.

PCR amplification proficiency test experiment (E2): All participants applied the PCR protocol (https://dx.doi.org/10.17504/protocols.io.bd94i98w) to amplify the 312 bp rbcL barcode (Vasselon et al. 2017) from four DNA calibrated samples: river biofilm (R), lake biofilm (L), mock community (M) and DNA free water (W) as negative control (Table 1). PCR amplifications were performed in triplicate for each sample using Takara LA Taq polymerase (Takara) in a final mix volume of 25 μl. Importantly, the reference laboratory supplied reagents (rbcL primers, Takara LA kits) for PCR amplification from the same lot to each participant. Amplification success was visually confirmed by participants using agarose gel electrophoresis and amplicon triplicates were pooled together to send 57 μl of each samplés amplicon to the reference laboratory for downstream HTS library preparation and sequencing. These instructions were given to the participants as described in Suppl. material 1: fig. S2.

### Methods comparison experiments

We made comparisons of methods related to DNA extraction (E3) and PCR amplification (E4) steps by asking participants to use their own choice of protocols on a set of calibrated samples. Nine of the 17 participants had an internal protocol different from the reference laboratory for E3 and E4 and, therefore, participated in these experiments. Detailed protocols were not collected and only key steps and characteristics were collected to maintain anonymity of participants (Suppl. material 1: tables S2, S3).

DNA extraction experiments (E3): These nine participants used their own choice of DNA extraction methods and protocols on the same calibrated samples used for E1 (R, L, M, W). As a result, a significant number of parameters were expected to vary amongst participants (e.g. the lysis method, the use of proteinase K and RNase A and the purification method, Suppl. material 1: table S2). DNA extractions were conducted in triplicate for each sample and participants were asked to send a minimum of 20 μl of DNA extract per sample replicate to the reference laboratory. These instructions were given to the participants as described in Suppl. material 1: fig. S3.

PCR amplification experiments (E4): Nine participants applied their own choice of PCR amplification protocol to amplify the 312 bp rbcL (263 bp barcode and the primers) on the same DNA calibrated samples used for E2 (R, L, M, W). As for E3, several parameters are expected to vary, especially according to the distinct polymerase used (e.g. exonuclease activity, hot start activity, sensitivity, fidelity, efficiency, Suppl. material 1: table S3). For each participant, three PCR replicates of 25 μl per sample were performed, each comprising 33 cycles, with an annealing temperature of 54 °C, to maximise comparability. Participants were asked to send to the reference laboratory a minimum of 60 μl of amplicons per sample replicates. These instructions were given to the participants as described in Suppl. material 1: fig. S4.

### Library preparation and HTS sequencing

The return shipments from all participants totalled 68 DNA extracts (4 samples, 1 replicate, 17 participants) from the E1 experiments and 108 DNA extracts (4 samples, 3 replicates, 9 participants) from the E3 experiment. The reference laboratory (RL) performed *rbc*L 312 bp PCR amplification on each DNA extract in triplicate, following the same protocol as described previously for the E2 experiments. PCR triplicates were then pooled together to obtain one pool of amplicon per sample. Additionally, a total of 68 pools of amplicons (4 samples, 1 pool replicate, 17 participants) from E2 experiment and 108 (4 samples, 3 pool replicates, 9 participants) from E4 experiment were also received. RL produced a total of 12 DNA extracts (4 samples, 3 replicates) corresponding to 12 pools of amplicons after PCR amplification. A total of 364 individual pools of amplicons were obtained from all experiments. Their quality was controlled using gel electrophoresis; negative controls (W) were not sequenced, except for 3 W samples (E1_E, E1_Q, E3_O) when unexpected amplification was observed (potential contamination). The amplicon quality of one sample from the E2 experiment (participant G, L sample) was considered to be too low (faint amplicon band in gel electrophoresis) and not sequenced.

Altogether, a total of 275 successful PCR reactions from E1, E2, E3 and E4 experiments were sent to the INRAE Transfert sequencing facility (Toulouse, France), which carried out the final library preparation for MiSeq pair-end (PE) 2*250 bp (from the PCR2 to the equimolar pooling of the amplicons). Three samples from the E4 experiment (2 replicates of M sample from participant P and 1 replicate of R sample from participant E), produced less than 1000 reads after bioinformatics treatments. In order to conserve the statistical design, they were sequenced in a second MiSeq PE 2*250 bp sequencing run. All the samples and replicates produced by the RL were included in both runs to evaluate potential variability introduced by the two runs prior validating the use of those three samples during downstream analysis.

### Sequencing data processing

A total of 9,369,334 raw reads were obtained from the first MiSeq PE sequencing runs along with a further 257,686 reads from the three samples from the second MiSeq PE run. The raw data consisted of demultiplexed fastq files pairs (R1.fastq and R2.fastq) per sample accessible on the NCBI Sequences Read Archive (SRA) under the BioProject accession numbers PRJNA1187555 for experiments E1 and E3 and PRJNA1187576 for E2 and E4. Bioinformatics treatments of raw MiSeq demultiplexed data were performed using DADA2 (Callahan et al. 2016) in R (R Core Team 2020) which enables Amplicon Sequence Variants (ASVs) to be inferred from DNA reads. We used an adjusted version of the pipeline for diatom DNA metabarcoding based on the *rbc*L 312 bp barcode, available on Github (https://github.com/fkeck/DADA2_diatoms_pipeline), using parameters described in Tapolczai et al. (2019). As two MiSeq runs were conducted, the error rates model determined by the DADA2 denoising algorithm will be different. To account for this, bioinformatics treatments were initially performed separately for each run until the denoising step. Then, the data from all samples were combined prior to the chimera removal step. Following the production of the ASVs table, taxonomic assignment of ASVs was achieved using the RDP classifier as implemented in Mothur (Schloss et al. 2009) (classify.seqs, wang algorithm, cutoff = 75, iter = 1000) combined with the Diat.barcode reference library V9 (Rimet et al. 2019, library available at https://doi.org/10.15454/TOMBYZ). ASVs identified as non-Bacillariophyta phylum or as Bacillaryophyta_unclassified were discarded. Finally, ASVs with less than 10 reads and representing less than 0.01% per sample were removed.

### Morphological data

Although the original objective was to perform morphological intercalibration experiments involving all participants using the same calibrated samples, major coordination challenges were encountered (see Discussion). Consequently, only the reference laboratory performed the morphological determination, to provide a first comparison of the IBD and IPS scores and the intra-operator variability obtained through this additional method following European and French standards (AFNOR 2016). Briefly, samples underwent successive baths of hydrochloric acid (HCl) and 40% hydrogen peroxide (H_2_O_2_) to remove organic matter and calcium carbonate, leaving only the silica valves of the diatoms. The resulting preparations were used to make three permanent slides per sample using resin (Naphrax^©^). A minimum of 400 valves were counted per slide and identification was performed with a microscope in accordance with European standards (EN 14407 2014) and using European floras (as described in Rivera et al. (2020)). The final taxonomic lists were expressed as the relative proportion of valves per diatom taxa.

### Data analysis of proficiency tests (E1 and E2)

Several key metrics were computed for all samples: DNA concentration [DNA] for E1, number of DNA reads and diversity metrics (of ASVs and Species) expressed with Hills numbers (q = 0, q = 1, q = 2) for both E1 and E2. Briefly, Hills numbers mathematically unify diversity concepts and are well adapted to comparison of DNA metabarcoding outputs (Mächler et al. 2021). A Hill number with an order: (q = 0) is analogous to species richness measure, (q = 1) is analogous to the exponential of Shannon diversity as an alpha diversity measure and (q = 2) is analogous to the inverse of the Simpson index. The Coefficient of Variation (CV) was subsequently determined to assess the degree of variability associated with these metrics across all participants. The resulting CV values were represented with radar charts.

The molecular and morphological inventories obtained from the river and lake samples were used to compare diatom community composition obtained by each participant in experiments E1 and E2. Additionally, the resulting molecular and morphological inventories were also used to compute the Specific Pollution Sensitivity index (IPS) (Cemagref 1982) and the Biological Diatom Index (IBD) (Coste et al. 2009). The IBD index is the benthic diatom index used for routine WFD river monitoring in France and the IPS index is widely used for European intercalibration studies (Kahlert et al. 2009, 2012). Both indices were originally developed for measuring the impact of water of rivers, not lakes, based on morphological diatom inventories. However, in this study, we utilised these indices not for ecological assessment, but to interpret variability amongst index scores and their associated ecological classes. IPS and IBD scores were calculated using OMNIDIA software version 6.0 (Lecointe et al. 1993).

In order to evaluate the performance and consistency of all participants (including RL) in the proficiency tests, z-scores were computed, based on the IPS scores for R and L samples. z-scores, also called “standard score”, show how far the IPS value of one participant is from the mean IPS of all participants and is calculated as follows: z=(x−μ)/σ where x is the IPS value of the participant, μ is the mean value of all participants and σ the corresponding standard deviation. Z-scores usually ranged from −3 SD to +3 SD. In the context of a proficiency test, any Z-score value < −3SD or > +3SD indicates that the protocol tested is not reproducible between the participants and has failed the proficiency test (ISO 13528 2015, see section “Calculation of performance statistic” and subsection “z scores” for more details).

As IPS z-scores were computed for each participant on two environmental samples (R, L), then they can be projected one to another as a Youden plot (Youden 1956). This graphical method allows within-laboratory variability as well as between-laboratory variability and estimates the systematic, random and total errors to be visualised.

### Data analysis of method comparison experiments (E3 and E4)

To evaluate the consistency of all participants in applying their own choices of DNA extraction and PCR protocols, the diatom assemblages obtained by each participant for each replicate were analysed using histograms, with ASVs gathered at the species level. Non-metric multidimensional scaling (nMDS) was used to assess variation in diatom composition both between and within participants visually. The nMDS was performed on Bray-Curtis distances of diatoms, based on relative abundance using the vegan package (Oksanen et al. 2019). In addition, significant differences both within participants, or within methods, were assessed through a permutational multivariate analysis of variance (PERMANOVA) followed by a multivariate pairwise comparison. These analyses were performed through the *adonis*() and *mrpp*() functions, respectively, with the Vegan package (Oksanen et al. 2019). As for experiments E1 and E2, IPS and IBD scores were also computed.

## Results

### Feedbacks from experiments

An online workshop was organised on 21 March 2021 to present preliminary results to the participants and collect feedback on the experiment. All participants successfully completed the experiments within the expected timeframe using the materials and protocols provided. Despite the application of new DNA extraction (E1) and PCR amplification (E2) protocols on blind calibrated samples, the feedback was highly positive in terms of their accessibility and operability, even for the molecular biology laboratories which defined themselves as “less experienced”. A wide range of methods were employed by individual laboratories for DNA extraction (E3) and PCR amplification (E4) (Suppl. material 1: table S3). Nonetheless, participants readily adjusted their protocols to the provided samples and the experimental conditions.

The quality and quantity of DNA extracted during experiments E1 and E3 were assessed by the participants using different methods and devices, hindering direct comparisons. Consequently, the reference laboratory quantified DNA extracts using a standardised method (Picogreen), except for one participant (E), who did not have any DNA available for quantification. Variability was observed in DNA concentration obtained by the 17 participants and the reference laboratory (n = 18) for the river, lake and mock samples with an average of 43.7 ng μl^−1^ (SD: ± 27.8), 25.2 ng μl^−1^ (SD: ± 12) and 1.2 ng μl^−1^ (SD: ± 1.6) respectively.

All PCR amplifications conducted during the E2 and E4 experiments were successfully completed by the participants, meeting the minimum quality requirements for *rbc*L amplicons set by sequencing facilities for MiSeq sequencing. However, during library preparation, unexpected amplification was observed in six of the negative controls (W samples). These samples were considered as “W false positive samples” and were consequently included in the MiSeq runs.

### Key molecular and morphological working tables

After bioinformatics filtering steps, a total of 275 samples representing a total of 3,029,482 DNA reads were retained, corresponding to: (i) the L, R and M biofilm samples sent to the participants for E1 (51) and E3 (81), (ii) the L, R and M DNA samples sent to the participants for E2 (50) and E4 (81), (iii) the samples prepared by the reference laboratory for comparison in all experiments (9) and (iv) the three “W false positive samples”. The composition of two “W false positive samples” were associated with E1 and corresponded exactly to a composition of an “L” sample, while the third one was associated with E3 and corresponded to an “R” sample. Consequently, these errors might be related to a pipetting error resulting to a mis-transferred sample in the W tubes, occurring before or after the DNA extractions. These errors were considered as external to the variability introduced during the protocol and, consequently, these “W false positive samples” were removed from the analysis. The resulting ASVs and corresponding molecular diatom species inventories, expressed as proportions of total DNA reads, were generated and used for downstream analyses (available on Zenodo repository system). Morphological diatom species inventories were produced by the reference laboratory (Suppl. material 1: table S1).

### Proficiency tests (E1 and E2)

**Figure 2.**
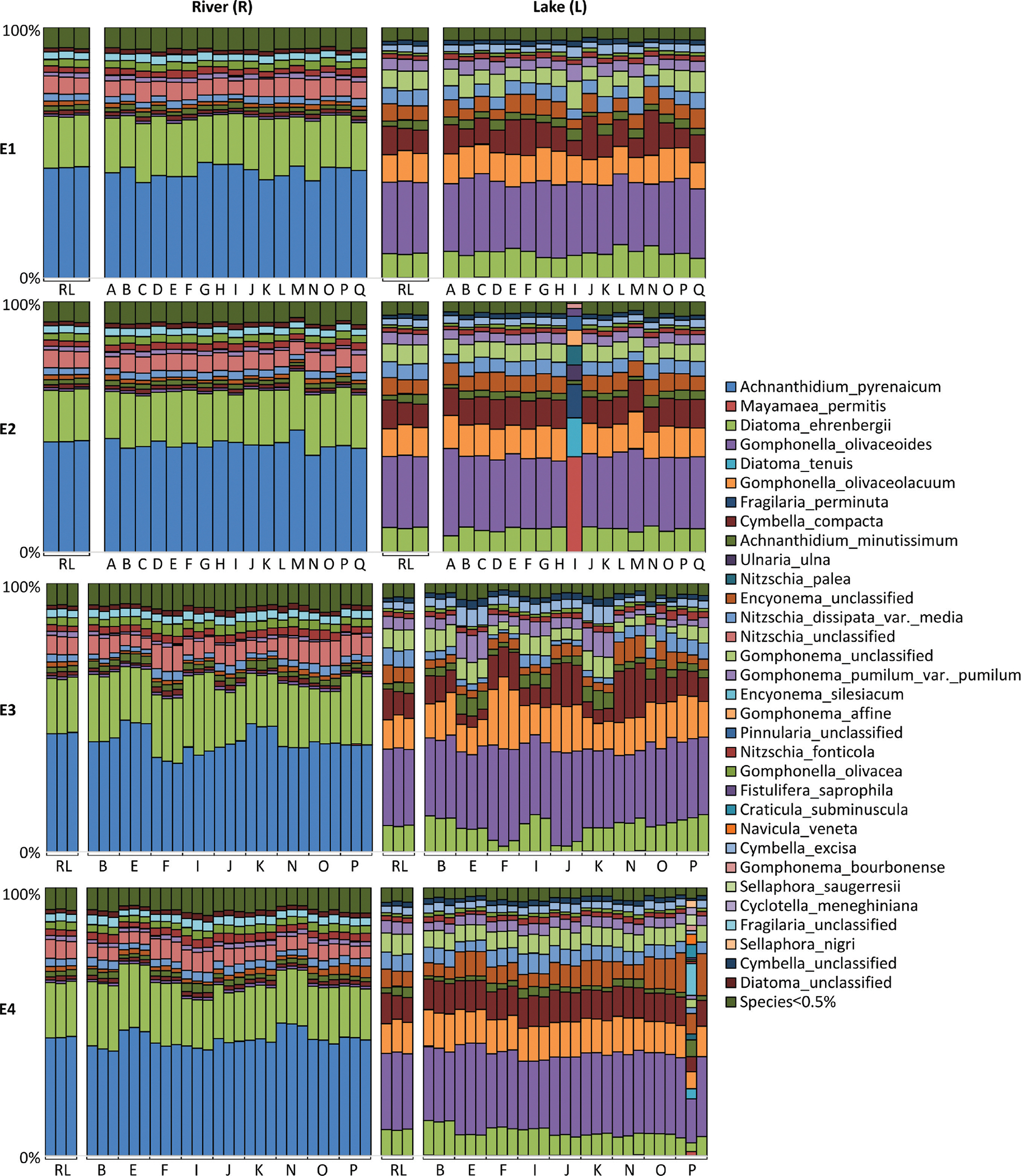
Histograms of the diatom community composition (at the species level), for each of the four experiments (E1 to E4). Sample labels correspond to participant codes. RL corresponds to the reference laboratory.

The diatom assemblage composition obtained from the river (R) and lake (L) biofilm samples showed high similarity amongst the 17 participants and the reference laboratory when using the same DNA extraction (E1) and PCR amplification (E2) protocols (Fig. 2).

However, one exception was observed for participant I (E2), where an unexpected diatom composition was obtained for the L sample, corresponding to a 1:1 mix of the L and M communities. As this non-systematic error was only detected from one participant in only one condition, it might occur during a transfer of sample, either during: (i) the sample aliquot preparation by the reference laboratory, (ii) the sample manipulation by the participant after the extraction or (iii) the HTS library preparation. Consequently, this error could not be related to the variability introduced during the application of the protocol within the participant environment (e.g. equipment, lab decontamination, manipulator practices, general contamination) as it is addressed during the proficiency test. As this “outlier” will affect the computation of z-score and the result of the proficiency test, this sample was excluded from subsequent analyses. When analysing the complete dataset, taxa with relative abundance per sample higher than 0.71% for E1 and 0.96% for E2 were successfully detected by all participants for both environmental samples (R, L). However, detection varied amongst participants for taxa below these abundance thresholds, indicating differences in their detection probability within the samples.

**Figure 3.**
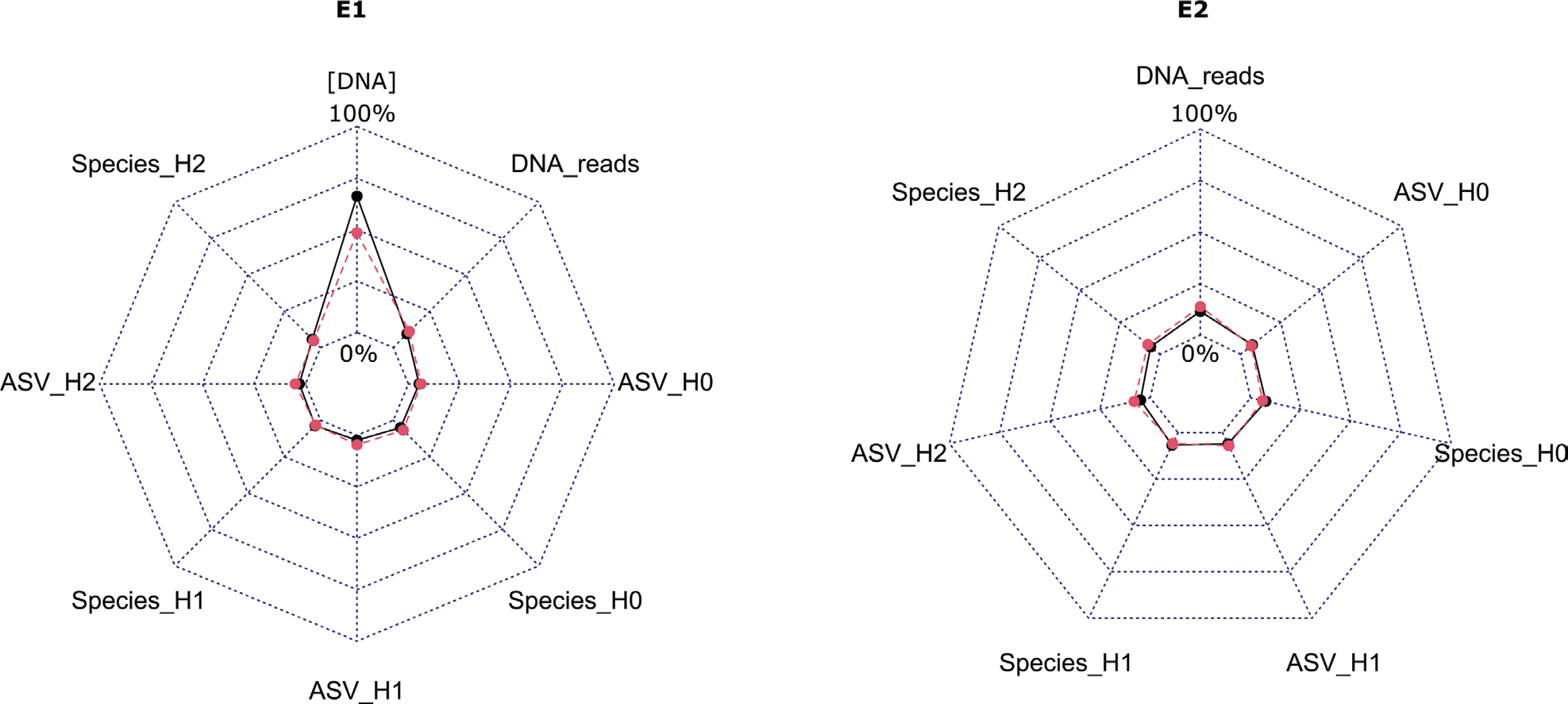
Radar charts presenting the coefficient of variations for the different metrics for the river “R” (Black line/dot) and lake “L” (Red line/dot) samples related to the Hill’s numbers (for E1 and E2), the DNA concentration (for E1) and the DNA reads numbers (for E2).

The concentration of DNA exhibited the highest CV amongst participants, with values of 66.2% for river samples and 48.5% for lake samples. However, for all other metrics, CV values were below 15% (Fig. 3).

**Figure 4.**
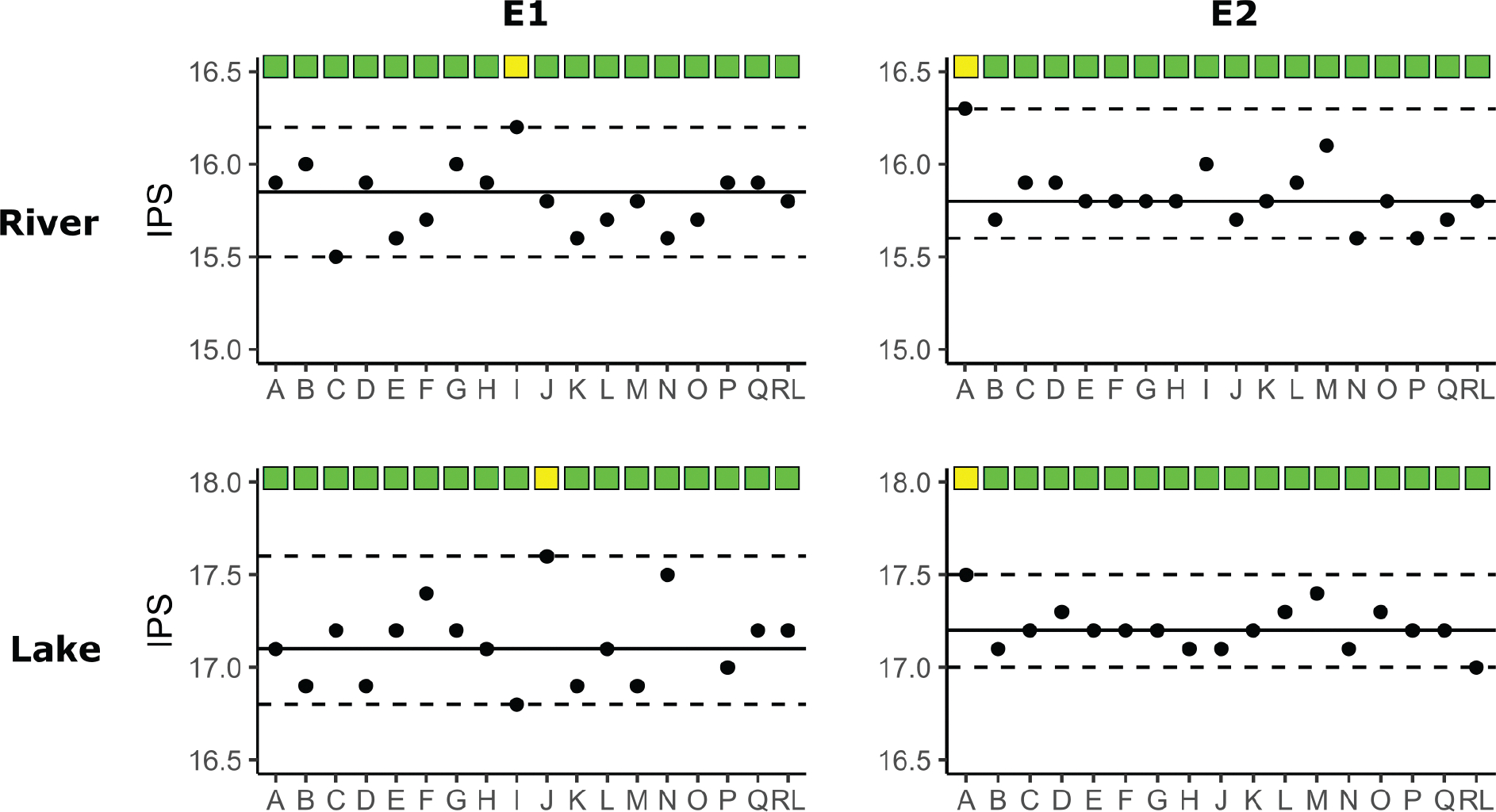
IPS scores obtained for E1 and E2 experiments calculated on the river and lake samples. Z-score were computed for each participant and condition, green box indicate a z-score between [− 2SD; + 2SD], while a yellow box indicated a z-score between [− 3SD; − 2SD] and [+ 2SD; + 3SD]. The proficiency test was considered successful as no participant had a z-score] − 3SD; + 3SD]. The RL was considered here as a participant and not as a validation reference for “true values”.

IBD and IPS scores were computed from the molecular taxonomic lists for E1 and E2 environmental samples. Consistent IBD scores of 20 (SD: 0) were obtained amongst all participants for both lake (L) and river (R) samples. The absence of variation in IBD scores between participants indicated a successful proficiency test for E1 and E2 experiments concerning this index. However, some variation was observed amongst IPS scores, with IPS deltas between participants of 0.8 for E1-lake (median: 17.1, SD: ± 0.21), 0.7 for E1-river (median: 15.9, SD: ± 0.17), 0.5 for E2-lake (median: 17.2, SD: ± 0.12) and 0.7 for E2-river (median: 15.8, SD: ± 0.16) (Fig. 4). Despite these variations, Z-scores computed for IPS scores fell within the accepted range of [−3;3] for the proficiency test (Fig. 4).

**Figure 5.**
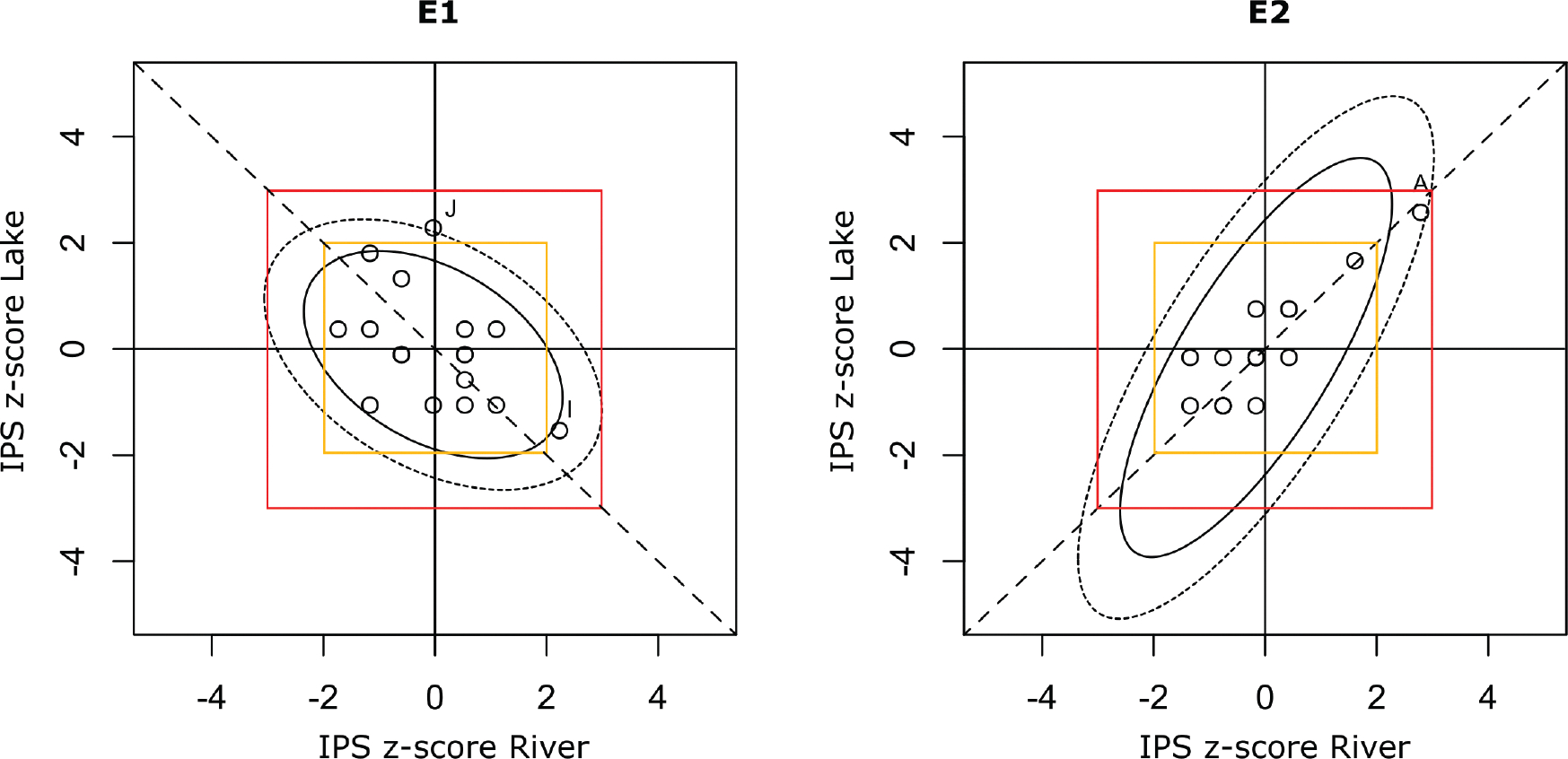
Youden plots performed with the IPS z-scores obtained for the river and lake samples. Red square highlights the 3 standard deviation (SD) limit and the yellow box the 2 SD limit. The 45-degree reference line helps to visualise if participants have a systematic error (point close to the reference line and outside the red box), total error (point far from the reference line and outside the 3 SD box) or a random error (point far from the reference line, but within the 3 SD box).

Youden plots were generated for the E1 and E2 experiments using IPS z-scores obtained for river (R) and lake (L) samples (Fig. 5). In the E1 experiment, a slightly elliptical distribution of participants was observed, suggesting that systematic error was slightly more pronounced than random error for two participants (I, J) falling outside the 95% probability range. Participant J was positioned closer to the y-axis indicating a significant bias associated with a total error occurring on lake sample, while participant I was situated along the 45 ° diagonal, suggesting a strong systematic error. In the E2 experiments, a clear elliptical distribution of participants was evident, indicating that a systematic error was more pronounced than a random error for one participant (A) outside the 95% probability range.

### Methods comparison experiments (E3 and E4)

The results obtained from the E3 (DNA extraction) and E4 (PCR method) experiments revealed a high degree of similarity in diatom assemblage composition amongst the technical replicates produced by the nine participants and the reference laboratory (Fig. 2). However, an exception was observed in the L sample of the E4 experiment, where participant P yielded an unexpected composition for one of the three replicates. This exception was likely due to a sample processing error, such as manipulation or contamination, prompting the removal of this replicate from further analysis. All participants were able to detect taxa with relative abundance per sample higher than 1.73% for E3 and 0.85% for E4 experiments in both environmental samples (R, L). However, taxa below these abundance thresholds exhibited unequal detection rates within and between participants, likely influenced by variations in their detection probability, the efficacy of their protocol or the stochastic effect within the samples.

**Figure 6.**
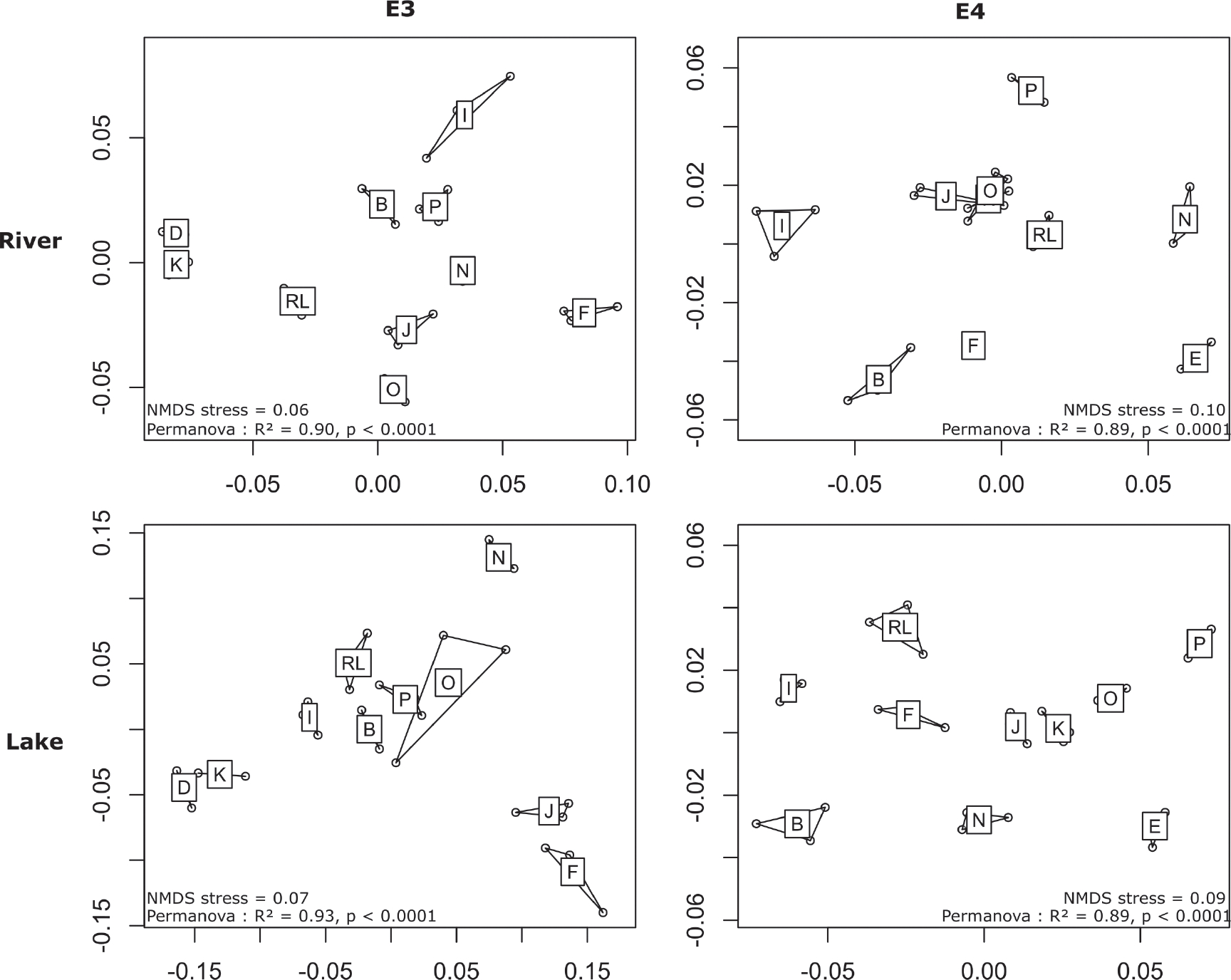
NMDS plots performed with the Bray-Curtis dissimilarity index calculated with the datasets obtained from both E3 and E4 experiments. Sample labels correspond to the participant codes. RL corresponds to the reference laboratory.

Comparison of diatom assemblage structures in E3 and E4 experiments was performed using Bray-Curtis dissimilarity indices. Between-participant variability remained low as depicted in the nMDS plot (Fig. 6), which is consistent with previous observations from community composition in Fig. 5. Within-participant variability remained consistent, with sample replicates clustering together as depicted in the nMDS plot (Fig. 6). For E3 experiments, the PERMANOVA test revealed that participant effects accounted for 90% (*p* < 0.0001) and 93% (*p* < 0.0001) of the total variance observed in river and lake samples, respectively (Suppl. material 1: table S4). Similarly, in the E4 experiments, PERMANOVA results attributed 89% (*p* < 0.0001) and 89% (*p* < 0.0001) of the total variance observed for river and lake samples, respectively, to participant effects (Suppl. material 1: table S5). As the participant replicates are highly repeatable, within-participant variability is lower than between-participant variability and the PERMANOVA test was able to detect highly significant differences from slight fluctuations in the community structure. Differences amongst participants were mainly due to differences in the detection of low abundant taxa (relative abundance < 1%) and in the quantification of abundant taxa (> 1%). For instance, in the E3 experiment, participants D and F had the highest dissimilarity between their community structure in the R sample (Fig. 6) due mainly to the high average relative abundances of *Achnanthidium pyrenaicum* of 48.3% and 33.9%, respectively, thereby accounting for a significant portion of the observed divergence in their assemblage structure.

As participants employed their own choice of DNA extraction (E3) and PCR amplification (E4) methods, we also explored the potential impact of these procedures on diatom assemblage structure through the nMDS and using a PERMANOVA test. In the E3 experiments, the diverse cell lysis methods incorporated in DNA extraction protocols, including enzymatic (river: 66.1%, lake: 72.2%) and mechanical (river: 11.4%, lake: 20%) approaches, yielded significant PERMANOVA results with respect to the total variance observed between samples (Suppl. material 1: fig. S5, table S6). In the E4 experiments, DNA polymerase characteristics (Suppl. material 1: table S3) as provided by the participants and the providers’ documentation were not considered comparable as the meaning of the terminology might vary between suppliers. For example, different terms such as “high efficiency”, “high fidelity” or “high sensitivity” are not clearly defined by the suppliers, in the absence of clear measurements and thresholds. Decipher variability introduced in our DNA metabarcoding data by DNA polymerase variability would require specific investigation, in a similar way as performed by Nagai et al. (2022); thus, no statistical analysis will be presented.

**Figure 7.**
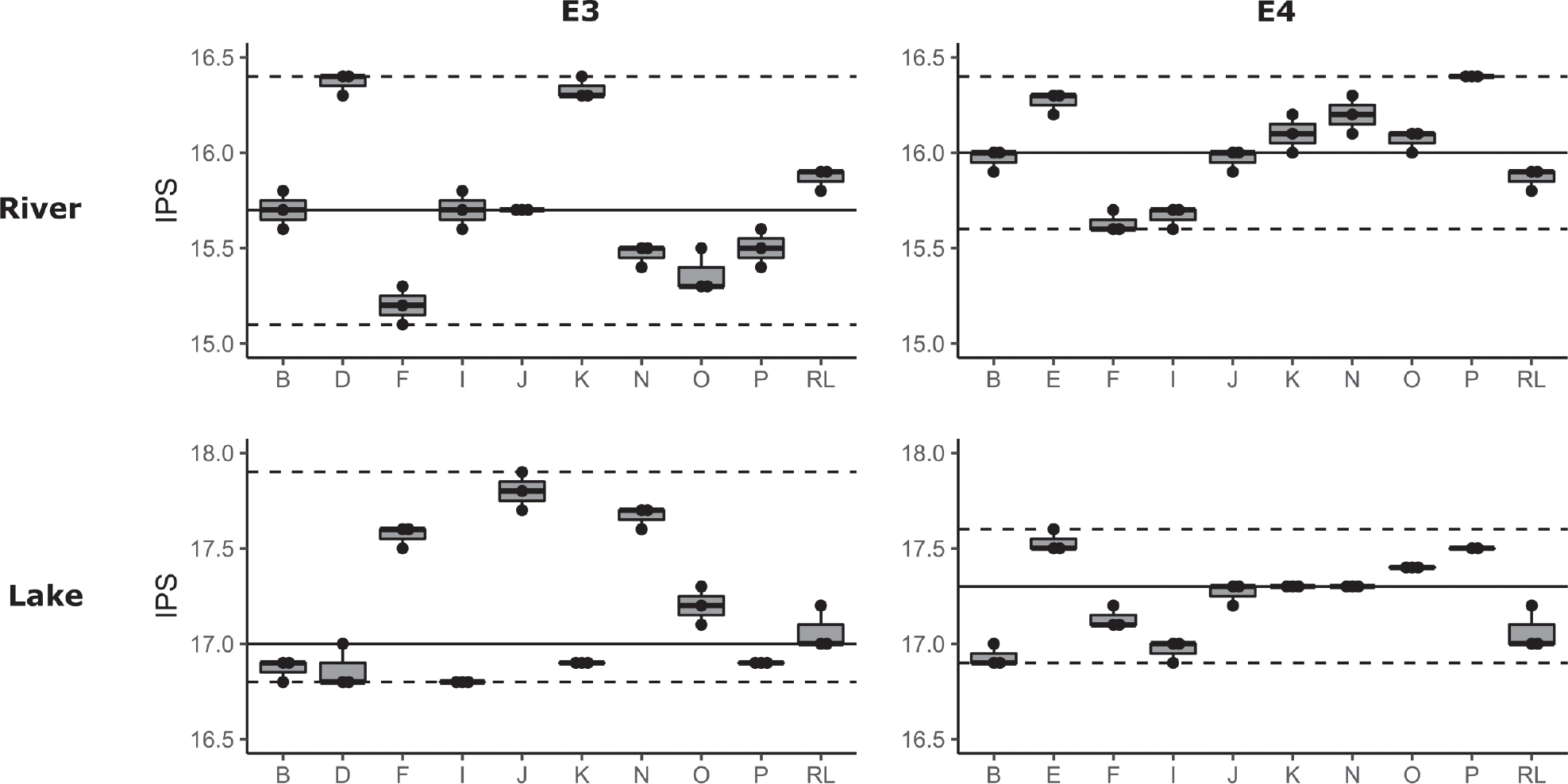
IPS scores obtained for E3 and E4 experiments on the river and lake samples for nine test laboratories (letters) and the reference laboratory (RL). The RL was considered here as a participant and not as a validation reference for “true values”.

Ecological assessment was performed by computing IBD and IPS scores using molecular taxonomic lists for E3 and E4 environmental samples. As for the E1 and E2 experiments, consistent IBD scores of 20 (SD: 0) were obtained for lake and river samples across all participants and replicates. However, in the E3 experiments, IPS scores exhibited significant variation amongst participants for both river (Kruskal-Wallis, *p* = 0.002) and lake (Kruskal-Wallis, *p* = 0.001) samples, with an IPS delta between participants of 1.1 for lake (median: 17, SD: ± 0.37) and 1.3 for river (median: 15.7, SD: ± 0.38) samples. Similarly, in the E4 experiments, IPS scores also displayed significant differences between participants for river (Kruskal-Wallis, *p* = 0.001) and lake (Kruskal-Wallis, *p* = 0.001) samples, with an IPS delta between participants of 0.7 for lake (median: 17.3, SD: ± 0.21) and 1.2 for river (median: 16, SD: ± 0.24) (Fig. 7). The within-participant variability (n = 3 replicates) of IPS scores was low, with an average of 0.1 for lake and river samples in E3 and E4 experiments.

### IBD and IPS morphological scores

Diatom taxa from both lake and river samples were also identified in triplicate by the reference laboratory using their morphological characteristics (Suppl. material 1: table S1). Ecological assessment was performed by computing IBD and IPS scores. For the R sample, the IBD score was consistently 20 (SD: 0) for all replicates, while IPS score averaged 18.7 (SD: ± 0.3 amongst replicates). The IBD score for the L sample averaged 19 (SD: ± 0.2), while the IPS averaged 15.9 (SD: ± 0.1).

## Discussion

### Validation of the proficiency tests shows that the applied DNA and PCR protocols can perform reproducible molecular diatom freshwater biomonitoring

Through this study, we first compared the results obtained from 17 participating laboratories using the same reference protocols for DNA extraction and PCR1. Regardless of their random and systematic errors, we observed a high consistency in the ecological assessments (IBD and IPS scores) for both experiments. While most participants developed their own protocols, they agreed to perform “blind” experiments with an agreed standard laboratory protocol. Thus, our proficiency test results demonstrate the potential transferability of a common DNA extraction and PCR amplification protocol, as reproducible results were obtained across different participants. Diatom molecular taxonomic assemblages also exhibited high consistency amongst participants for the three calibrated samples, despite an unequal detection of taxa of low abundance (< 1%). For ecological assessment purposes, this is acceptable as IPS and IBD scores (and, indeed, most other metrics in common use) are primarily driven by taxa with proportions exceeding 5% (Bigler et al. 2010). This highlights the need to clearly define the application parameters of the genetic method, both for intercalibration and standardisation purposes, as performing DNA metabarcoding for ecological assessment and for characterisation of “rare” taxa may require different strategies (replicates, sufficient sequencing depth).

The coefficient of variation (CV) values underscores the stability of most metrics, except for the DNA concentration during the E1 experiment, which exhibited high variability. Variation in DNA concentration from the same sample and protocol within a participating laboratory may arise due to technical factors, such as pipetting inconsistencies and equipment variations, sample heterogeneity, potential contamination and inherent biological variability in cell abundance or extracellular DNA content (Greathouse et al. 2019). These factors emphasise the need for robust quality control measures and replication to mitigate variability and ensure consistency in molecular biology experiments. Despite the high variability in DNA concentration, PCR amplifications were successful and did not limit the interpretation of the diatom DNA metabarcoding results based on assemblage composition and final assessment outcome. Nevertheless, an exception was noted with participant I (E2), where an unexpected diatom composition was observed. This anomaly underscores the importance of stringent quality control measures, as deviations may arise from sample processing errors or contamination, emphasising the need for replicates and thorough validation procedures to ensure result integrity (Robasky et al. 2014). In combination with negative controls and reference sample (e.g. defined mock community, synthetic DNA templates, calibrated environmental sample), quality control should be considered at each major step of the metabarcoding process based on internal process controls (IPCs): DNA extraction (DNA quality and quantity, biofilm biomass, level of inhibition), PCR amplification (barcode and primer choice, technical replication, number of PCR cycles, amplification success, level of non-specific amplification), HTS sequencing (platform and sequencing kit chemistry choice, sequencing depth) and bioinformatics (software and pipelines accepted, post analysis sequencing depth, DNA reads loss per bioinformatics step, negative control handling). However, a balance between quality stringency and implementation will have to be found in the context of freshwater biomonitoring as environmental samples and methods used may introduce variability without impact on final assessment. As an example, our study showed high variability in DNA concentrations obtained by participants depending on DNA extraction protocol choice, yet this variation had limited influence on the final IPS score. Thus, DNA extract quality and quantity should be kept as an IPC to validate the success of the DNA extraction, but even for low quantity and quality samples, biomonitoring using short read metabarcoding can work reliably. As numerous data are available on diatoms, dedicated work should be performed to highlight key quality control to consider and their operational threshold for biomonitoring.

The high degree of similarity observed amongst participants and the reference laboratory in diatom assemblage composition when using identical DNA extraction and PCR amplification protocols (E1 and E2) attests to the reliability and consistency of these protocols, reinforcing their reproducibility. This suggests that standardised protocols can form a foundation for inter-laboratory consistency in diatom analysis. Despite the controlled conditions of the proficiency tests, with calibrated samples, identical protocols and reagents, not all sources of variability could be evaluated (operator, machines, participant lab environment etc.). Source of variability within eDNA studies can be highly diverse and, thus, difficult to predict as environmental characteristics might change from one waterbody to another, affecting eDNA approaches (Stoeckle et al. 2017; Mathieu et al. 2020). In the context of the WFD where around 115,000 rivers and 25,000 lakes sites are monitored around the EU (WISE, https://water.europa.eu/freshwater), the diversity of environments and conditions will be difficult to predict. In order to meet the ISO requirements, it was logistically feasible to include only one lake and one river sample in E1 and E2 experiments (ISO 13528 2015, 2022). While this enables the validation of method stability and transferability of the DNA extraction and PCR amplification protocols used, our experiments alone are not sufficient to validate the protocols for use on large-scale monitoring networks with a wide variety of rivers. This is why we would recommend additional intercalibration studies on other river and lake types.

Factors affecting test reproducibility amongst laboratories can be related to laboratory environment, biases introduced by human technical practices, model of instrument and calibration (Waugh 2021). Those biases might be negligible, but they are unfortunately difficult to detect and to correct. Our intercalibration study, through the identification of systematic and random errors using z-score and Youden plot projection, allowed participants to compare their results with others, but also highlighted potential deviation in internal laboratory processes. Several PCR amplification biases are known to affect DNA metabarcoding data (e.g. primer biases, polymerase capabilities) and several studies have documented strategies to minimise their effect (Krehenwinkel et al. 2017; Nichols et al. 2018; Moinard et al. 2023). However, little information is available regarding laboratory technical biases (e.g. lab practices, thermal cycling machine deviation, reagents storage, plastic-ware used). This might explain why some participants produced IPS scores for both samples that systematically deviated from results produced by the group during PCR amplification experiments (e.g. E2, participant A). As taxonomic composition and IPS scores obtained were consistent with other participants, the systematic error produced had barely detectable downstream effects. The use of proficiency tests enables the detection of such systematic errors, aiding laboratories in investigating and improving their practices. We also observed total error in E2 with participant I who produced highly divergent diatom assemblage composition and was excluded from downstream analyses. The use of technical replicates can easily help to detect such errors allowing the elimination of any erroneous replicates, as observed in E4 with participant P on the lake with the removal of the 2^nd^ replicate.

Finally, the proficiency test we conducted was focused on evaluating DNA extraction and PCR amplification protocols. While these steps are crucial and our results validate the potential to transfer laboratory protocols, it is important to acknowledge that there are other key stages in the metabarcoding workflow that are known to introduce variability. These include library preparation methods (Zizka et al. 2019; Bohmann et al. 2022), sequencing technologies (Cuber et al. 2023), genetic markers (Kezlya et al. 2023), reference libraries (Keck et al. 2023) and bioinformatics pipelines (Bailet et al. 2020; Rivera et al. 2020). Therefore, similar experiments assessing these aspects are necessary to comprehensively understand and address sources of variability in diatom metabarcoding, facilitating the establishment of standardised protocols. As we showed in this study that intercalibration exercises for eDNA approaches can be easily organised, we would recommend to increase the efforts to organise both national (to integrate national particularities, for example, diatom index, methods, accreditation, legislation) and international exercises (to validate international inter-comparability of the data produced, support international standardisation process, follow technological and methodological evolutions). Such events should be opened to all sectors and institutions (research, industry and other public and private sectors). As DNA approaches are aimed to be compatible with WFD, we need standards and, thus, automatically an accreditation system will be necessarily developed to validate their correct application in routine monitoring (e.g. COFRAC in France related to AFNOR). However, such considerations should first be addressed at national level, as they can be country-specific.

### “All for one, but prove them all”: establishing proficiency testing schemes for performance-based quality management of DNA-based biomonitoring

The results of our proficiency experiments E1 and E2 showed that the implementation of a unique standardised molecular biology protocol can be easily achieved. Although this “One for all” strategy would favour the harmonisation of diatom assessments within freshwater monitoring programmes at the European scale as only one protocol would be used, it is not recommended for several sociological, economic and political aspects (Blancher et al. 2022; Kelly et al. 2024; Stein et al. 2024; Lee et al. 2024). One of the major risks highlighted in Lee et al. (2024) is the danger of technological “lock-in”, resulting in dependence regarding equipment, consumables and reagents. eDNA methods are evolving faster than our ability to standardise (Kelly 2019), thus a more integrative approach should be used. If several protocols are available and, despite their inherent variability and biases, produce similar results regarding diatom ecological assessment, they should be all acceptable for routine monitoring. This is in line with the “All for one” strategy that was validated within our E3 and E4 inter-comparison experiments.

Variations in the relative proportions of certain diatom taxa depending on the extraction protocol were previously highlighted by Vasselon et al. (2017). They demonstrated that the efficiency of the lysis method (e.g. mechanical, enzymatic or thermal) in breaking diatom cells can lead to variability in the relative abundance of specific diatom taxa in metabarcoding outputs. In our study, despite the low variability between participants regarding diatom community structure, the “participant effect” accounted for around 90% of the total variance within both samples and for each experiment, confirming these previous results (Vasselon et al. 2017). However, it is important to note that these variations did not significantly affect ecological assessments for the two samples we tested, as the overall composition of diatom assemblages remained consistent. The results obtained in experiments E3, where each participant used their own DNA extraction protocol, are consistent with these findings. Given these findings, it is likely that a combination of mechanical and enzymatic lysis mechanisms to break down the silica cell wall of diatoms and extract their DNA could lead to a more efficient extraction process, potentially reducing the variability associated with the diatom frustule issue. Further experimentation and downstream analysis are required to explore this possibility. Other studies testing different extraction methods conducted on other communities within biofilms from aquatic environments also demonstrated the importance of testing distinct lysis methods (Ferrera et al. 2010; Corcoll et al. 2017), considering that excessive mechanical treatments can result in the shearing of the DNA, decreasing its quality (Hermans et al. 2018; Portas et al. 2022).

The variability in taxonomic lists introduced by different PCR amplification protocols was surprisingly low in E4, considering the known biases associated with this technique. Our objective was to evaluate the potential effect of different PCR amplification protocols on diatom IBD and IPS indices and not to decipher all the variability within ASV or taxonomic data. Further analysis is needed to determine whether the observed biases are attributable to the specific Taq polymerase or thermal cycler used, as these factors may also contribute to variability (Dabney and Meyer 2012; Pan et al. 2014; Brandariz-Fontes et al. 2015; Nichols et al. 2018). The analysis of our metabarcoding data with dedicated metrics, such as proportions of chimera and introduced mutation (deletion, insertion, base substitution), would be of interest to evaluate the impact of the different DNA Taq polymerase applied by the participants (Nagai et al. 2022).

Results of our inter-comparison E3 and E4 experiments showed that there is a clear influence of participants and methods on the overall diatom assemblage structure, although the general composition remains consistent. Despite significant variations in IPS scores amongst participants, the observed range of variability falls within acceptable limits compared to typical variability observed in morphological intercalibration studies (Kahlert et al. 2012; Werner et al. 2016). Thus, the use of different DNA extraction and PCR amplification protocols yields consistent IBD and IPS results, indicating the operational viability of various approaches for diatom freshwater monitoring and the pertinence of the “all for one” strategy to support standardisation. In such a case, while each country may continue to use its own protocol or switch to new one following methodological innovation over time, it is important to implement harmonised quality control measures and minimum standard considerations to ensure reproducibility and to minimise variability. In line with this change of paradigm, we recommend the “all for one, but prove them all” strategy suggesting that new protocols can be used as long as the results are proved to be consistent with already validated protocols during intercalibration and inter-comparison experiments. These validation steps would: (i) guarantee the comparability of results, (ii) avoid lock-in risk by maintaining the use of different and independent protocols, (iii) give the freedom to see protocols appear or disappear at the same speed as technological innovations, (iv) stabilise standards and regulatory measures (training, accreditation) over time and (v) stimulate scientific innovation within the scientific community.

### Intercalibration and inter-comparison exercises as tools for integration of DNA approach along morphology for freshwater routine monitoring

The morphological approach remains the standard method for diatom identification in monitoring applications such as the WFD. However, this method requires significant time and expertise from highly-trained analysts and can result in significant variation in metric scores between operators (Kahlert et al. 2012; Werner et al. 2016). This inter-operator variability is mainly attributed to analyst-induced variance related to the difficulty of identifying some morphologically similar diatoms, the consistency of taxonomical expertise between operators and the natural heterogeneity of samples. As for many other microorganisms, diatom taxonomy is still not resolved since most taxa remain undescribed, some not separable based on LM criteria like for “cryptic” species and several decades of intense research efforts would be necessary to fill the gap (Mann and Vanormelingen 2013; Guiry 2024). Taxonomy is in constant evolution as more and more is known about their ecology, physiology, sexual reproduction and genetics favouring delineation of species (e.g. Ruck et al. (2016); Adl et al. (2019); Heudre et al. (2021); Mann and Trobajo (2024)). Results of river quality assessment, based on microscopy, can be quite different between operators, varying, for example, between 12 and 20 for the IBD index in one previous intercalibration exercise (Prygiel et al. 2002). However, despite these challenges, diatom indices are reliable for routine monitoring of river quality within the WFD (Kelly et al. 2009; Almeida et al. 2014; Kelly et al. 2014) and water managers have been using these tools for decades in Europe and America.

Morphological intercalibration exercises are already being performed in Europe (Kahlert et al. 2012 Werner et al. 2016). Such exercises are often organised between countries or at the national scale reflecting organisational as well as ecological differences around Europe (Kahlert et al. 2016) and involve participants from different backgrounds (authorities, consultants, researchers), frequencies (every year or occasionally), with more or less participants and the results of such events are generally published as “grey literature”. As organisation of such exercises is complicated even within a single country, their organisation at an international level is rare. Within our intercalibration exercises, R, L and M samples were prepared with the intention to perform morphological intercalibration experiments along with the DNA ones (data not shown). However, a major coordination challenge for European researchers was raised for such experiments which required: (i) adjusting the diatom flora used for the determination, (ii) increasing the number of valves identified (400 to 1000) to increase the depth of analyses and (iii) performing several replicates per sample which was considered highly time-consuming. Therefore, only the RL performed the morphological determination and showed that the variability within operators was at the same scale as inter-operator variability during DNA metabarcoding experiments. This result seems logical as it is easier to harmonise molecular methods and conditions between molecular ecology laboratories than harmonise taxonomical knowledge between taxonomists. Intercalibration exercises are necessary for morphological identification exercises in order to harmonise knowledge between operators and are necessary to improve national surveys (Kahlert et al. 2009; Kelly 2013). In several respects, coordination of DNA metabarcoding intercalibration and inter-comparison at the European scale may be easier than for morphological identification. However, integration of diatom DNA approaches along with morphological ones for freshwater assessment has been proposed by the scientific community (e.g. Charles et al. (2021)). This means that it may be necessary to build clear bridges facilitating intercalibration and inter-comparison experiments involving DNA and morphological approaches at the same time and not as separate events. As the organisation of morphological intercalibration exercises can be complicated, new propositions should be found to facilitate their organisation. For example, such efforts could be dedicated to easily connect diatomist experts (e.g. Spaulding et al. (2021)) or using digitalised diatom image (Kloster et al. 2020) to propose online exercises to diatomists as planned in the DNAquaIMG project (https://dnaquaimg.eu).

If intercalibration and inter-comparison exercises are essential parts of routine monitoring to maintain data continuity and consistency between future routine operators, clear guidelines need to be defined to coordinate the integration of molecular and morphological approaches at a national scale. Several documents propose practical guides on the use of eDNA approaches for freshwater monitoring (e.g. Pawlowski et al. (2020); Bruce et al. (2021); Blancher et al. (2022); De Brauwer et al. (2023)). If general documents are necessary to move forward with the integration of eDNA approaches, routine monitoring requires adapted standards for particular taxonomic groups, together with specific ecological and regulatory situations. One route for this is via international standardisation - via CEN and ISO as highlighted for eDNA diatom ecological assessment with technical reports for a routine sampling of benthic diatoms from rivers and lakes adapted for metabarcoding analyses (CEN/TR 17245 2018) and the management of diatom barcodes (CEN/TR 17244 2018). However, key performance standards and guidelines need to be produced on other steps of DNA metabarcoding to address operational questions on parameters, such as the number of sampling replicates, the use of calibrated samples (negative and positive controls including mock communities), the lysis method for DNA extraction, the strategy of number of PCR replicates per sample and subsequent pooling and the minimum copy number for retained sequences should be considered (e.g. Alberdi et al. (2018); Bunholi et al. (2023)). Consequently, further studies are required to provide more insight into the details of these guidelines, especially to provide estimation of the different thresholds required.

If results from our intercalibration and inter-comparison experiments is to contribute to the development of standards, a first priority would be to decide how to manage the integration of diatom quantification into diatom indices. As an example, several strategies were proposed to handle diatom quantification through metabarcoding using species correction, based on diatom cell biovolume (Vasselon et al. 2018) or a direct quantitative adjustment of the diatom index, as developed for the TDI (Kelly et al. 2020b). Such strategies have advantages, but also biases, which might directly affect routine monitoring like for IBD calculation where a percentage of contributing taxa should be reached within an inventory before validating the interpretation of the IBD (AFNOR 2016). However, despite discussing the upgrade of morphologically based standards to consider the variability of DNA corrected and non-corrected IBD scores, DNA metabarcoding data should also be considered independently of microscopy as discussed in Kelly et al. (2020a). This will help to create new metrics (Apothéloz-Perret-Gentil et al. 2017, 2021; Tapolczai et al. 2019; Kelly et al. 2020b) that can be used for freshwater biomonitoring and for which dedicated standards could be constructed without trying to fit with standards designed for microscopy-based indices. Once done, strategies that involve the co-application of DNA and morphology-based approaches taking advantage of both approaches could be efficiently designed and applied for large-scale monitoring.

## Conclusion

These intercalibration (proficiency test) and inter-comparison experiments demonstrate that diatom DNA metabarcoding offers a highly reproducible ecological assessment if the main taxa are included in the reference sequence database. In the experiments, participants used both a standard and an own-choice protocol and performed DNA extractions and PCR on calibrated samples. Congruent results (similar composition and indices) were obtained between laboratories, revealing: (i) the robustness of DNA extraction and PCR protocols between laboratories and (ii) the possibility of using different protocols (i.e. with different reagents), adding more variability to the assemblage composition, but without affecting the outcome of ecological assessment of the samples. As a result, we propose the “all for one, but prove them all” concept, suggesting that many different molecular approaches can be used as long as their functionality can be validated with the approach provided here (calibrated samples, negative and positive controls) and if key minimum steps (to be defined for future standards) are followed. Thus, our study shows how diatom metabarcoding can be included into routine monitoring of freshwaters in a regulatory context.

## Supplementary Material

Supplement1

## Figures and Tables

**Table 1.** Experimental design of the different experiments.

Objective	Experiment	No. of protocols	No. of participants	No. of replicates	Calibrated samples				
					Type	Lake (L)	River (R)	Mock (M)	C- (W)
**Proficiency test**	E1 - DNA extraction	1	17	1	Biofilm	1	1	1	1
	E2 - PCR amplification	1	17	1	DNA	1	1	1	1
**Method comparison**	E3 - DNA extraction	9 + RL	9	3	Biofilm	3	3	3	3
	E4-PCR amplification	9 + RL	9	3	DNA	3	3	3	3
**Reference**	E1, E2, E3, E4	1	1 (RL)	3	Biofilm	3	3	3	3

RL: Reference Laboratory. C - control, W – water.

## Data Availability

Raw reads were deposited and are publicly available in the NCBI Sequences Read Archive (SRA) under the BioProject accession numbers PRJNA1187555 for experiments E1 and E3 and PRJNA1187576 for E2 and E4. Complementary data are available on Zenodo online repository system using this link: https://zenodo.org/doi/10.5281/zenodo.12708767.
