# Supplementary material for: Proficiency testing and cross-laboratory method comparison to support standardisation of diatom DNA metabarcoding for freshwater biomonitoring
Source: Metabarcoding Metagenom. Author manuscript; Available in PMC 2026 Jan 10. (PMC11980862; doi:10.3897/mbmg.9.133264)

Supplementary information for:

Proficiency testing and cross-laboratory method comparison to support standardisation of diatom DNA metabarcoding for freshwater biomonitoring

Vasselon Valentin; Rivera Sinziana F.; Ács Éva; Almeida Salomé FP; Andree Karl B.; Apothéloz-Perret-Gentil Laure; Bailet Bonnie; Baričević Ana; Beentjes Kevin K.; Bettig Juliane; Bouchez Agnès; Capelli Camilla; Chardon Cécile; Duleba Mónika; Elersek Tina; Genthon Clémence; Jablonska Maša; Jacas Louis; Kahlert Maria; Kelly Martyn G.; Macher Jan-Niklas; Mauri Federica; Moletta-Denat Marina; Mortágua Andreia; Pawlowski Jan; Pérez-Burillo Javier; Pfannkuchen Martin; Pilgrim Erik; Pissaridou Panayiota; Rimet Frédéric; Stanic Karmen; Tapolczai Kálmán; Theroux Susanna; Trobajo Rosa; Van der Hoorn Berry; Vasquez Marlen I.; Vidal Marie; Wanless David; Warren Jonathan; Zimmermann Jonas; Paix Benoît

# Supplementary tables

## **Table S1.** Morphological diatom taxonomic list for the Lake (L), the River (R) and the Mock community (M) samples. Morphological identification was performed by the Reference Laboratory (RL) with 3 replicates per sample.

| **Species** | **Code** | **L1** | **L2** | **L3** | **M1** | **M2** | **M3** | **R1** | **R2** | **R3** |
| --- | --- | --- | --- | --- | --- | --- | --- | --- | --- | --- |
| *Achnanthidium minutissimum (Kützing) Czarnecki var. minutissimum* | ADMI | 8.22 | 9.05 | 9.09 | 8.09 | 1.45 | 4.99 | 2.41 | 2.25 | 1.99 |
| *Amphora pediculus (Kützing) Grunow var. pediculus* | APED | 1.64 | 2.44 | 1.47 |  |  |  | 0.48 | 0.25 |  |
| *Cocconeis pediculus Ehrenberg* | CPED | 0.23 |  |  |  |  |  | 0.24 |  |  |
| *Cocconeis placentula var. lineata (Ehrenberg)Van Heurck* | CPLI | 0.23 |  |  |  |  |  |  |  | 0.25 |
| *Cocconeis pseudolineata (Geitler) Lange-Bertalot* | COPL | 0.23 | 0.49 |  |  |  |  |  |  |  |
| *Cyclotella costei Druart & Straub* | CCOS | 0.23 | 0.24 |  |  |  |  |  |  |  |
| *Cymbella compacta Østrup* | CCMP | 10.09 | 9.29 | 11.55 |  |  |  |  | 0.25 | 0.5 |
| *Cymbella excisa Kützing var. excisa* | CAEX | 6.57 | 5.13 | 7.37 |  |  |  |  |  |  |
| *Diatoma ehrenbergii Kützing* | DEHR | 0.94 | 0.73 | 1.97 |  |  |  | 2.17 | 0.75 | 4.22 |
| *Encyonema minutum (Hilse in Rabh.) D.G. Mann in Round Crawford & Mann var. minutum* | ENMI | 4.46 | 12.71 | 8.11 |  |  |  |  | 0.5 |  |
| *Encyonopsis microcephala (Grunow) Krammer var. microcephala* | ENCM | 7.98 | 6.6 | 4.18 |  |  |  |  |  |  |
| *Gomphonema angustatum (Kützing) Rabenhorst var. angustatum* | GANG | 0.23 |  |  |  |  |  |  |  |  |
| *Gomphonema olivaceolacuum (Lange-Bert. & Reichardt) Lange-Bert. & Reichardt* | GOLL | 39.44 | 37.41 | 38.33 |  |  |  |  |  |  |
| *Gomphonema pumilum (Grunow) Reichardt & Lange-Bertalot var. pumilum* | GPUM | 4.46 | 4.89 | 5.41 |  |  |  | 0.24 | 2.5 | 0.99 |
| *Navicula cryptotenella Lange-Bertalot var. cryptotenella* | NCTE | 0.7 | 0.73 | 1.97 |  |  |  | 0.96 |  | 0.99 |
| *Navicula cryptotenelloides Lange-Bertalot var. cryptotenelloid* | NCTO | 1.88 | 0.73 | 2.46 |  |  |  |  |  |  |
| *Navicula gregaria Donkin var. gregaria* | NGRE | 0.7 |  |  |  |  |  |  |  |  |
| *Navicula reichardtiana Lange-Bertalot var. reichardtiana* | NRCH | 0.47 |  |  |  |  |  |  |  |  |
| *Navicula tripunctata (O.F.Müller) Bory var. tripunctata* | NTPT | 0.47 | 0.49 | 0.25 |  |  |  | 1.2 | 0.5 | 0.25 |
| *Nitzschia dissipata var. media (Hantzsch) Grunow in Van Heurck* | NDME | 7.98 | 2.69 | 2.46 |  |  |  |  |  |  |
| *Nitzschia dissipata subsp. dissipata (Kützing) Grunow var. dissipata* | NDIS | 0.23 |  |  |  |  |  | 4.82 | 3 | 3.72 |
| *Nitzschia fonticola Grunow in Cleve et Möller var. fonticola* | NFON | 1.88 | 3.42 | 1.97 |  |  |  | 2.41 | 3 | 2.48 |
| *Sellaphora nigri (De Not.) C.E. Wetzel et Ector comb. nov. emend.* | SNIG | 0.47 |  | 0.49 |  |  |  |  |  |  |
| *Staurosirella pinnata (Ehrenberg) Williams&Round var. pinnata* | SPIN | 0.23 |  | 0.98 |  |  |  |  |  |  |
| *Cocconeis pellucida Grunow ex Hantzsch in Rabenhorst var. pellucida* | CPEL |  | 0.49 |  |  |  |  |  |  |  |
| *Fragilaria perminuta (Grunow) Lange-Bertalot* | FPEM |  | 0.73 |  |  |  |  |  |  |  |
| *Nitzschia lacuum Lange-Bertalot* | NILA |  | 1.22 | 0.49 |  |  |  |  |  |  |
| *Nitzschia sociabilis Hustedt* | NSOC |  | 0.49 | 0.49 |  |  |  |  |  |  |
| *Cocconeis placentula Ehrenberg var. placentula* | CPLA |  |  | 0.49 |  |  |  |  |  |  |
| *Encyonema prostratum (Berkeley) Kützing* | EPRO |  |  | 0.49 |  |  |  |  |  | 0.5 |
| *Diatoma tenue Agardh var. tenue* | DITE |  |  |  | 12.75 | 9.93 | 7.36 |  |  |  |
| *Fistulifera saprophila (Lange-Bertalot & Bonik) Lange-Bertalot* | FSAP |  |  |  | 3.92 | 6.05 | 3.8 |  |  |  |
| *Fragilaria capucina var. vaucheriae (Kützing) Lange-Bertalot* | FCVA |  |  |  | 31.13 | 29.54 | 46.08 |  |  |  |
| *Fragilaria ulna sippen angustissima (Grunow)Lange-Bertalot* | FUAN |  |  |  | 5.88 | 3.15 | 1.43 |  |  |  |
| *Gomphonema affine Kützing var. affine* | GAFF |  |  |  | 2.21 | 1.45 | 0.95 |  |  |  |
| *Gomphonema bourbonense E. Reichardt et Lange-Bertalot* | GBOB |  |  |  | 0.49 | 2.18 | 2.38 |  |  |  |
| *Mayamaea atomus var. permitis (Hustedt) Lange-Bertalot* | MAPE |  |  |  | 22.06 | 37.53 | 23.75 |  |  |  |
| *Nitzschia palea (Kützing) W.Smith var. palea* | NPAL |  |  |  | 4.41 | 1.45 | 2.85 |  |  |  |
| *Pinnularia lundii Hustedt var. lundii* | PLUN |  |  |  | 2.7 | 3.87 | 4.99 |  |  |  |
| *Ulnaria ulna (Nitzsch) Compère var. ulna* | UULN |  |  |  | 6.37 | 3.15 | 1.43 |  |  |  |
| *Pinnularia viridiformis morphotype 1 Krammer* | PVIF |  |  |  |  | 0.24 |  |  |  |  |
| *Achnanthidium lineare W.Smith* | ACLI |  |  |  |  |  |  | 0.24 | 1.25 | 0.74 |
| *Achnanthidium pyrenaicum (Hustedt) Kobayasi* | ADPY |  |  |  |  |  |  | 74.46 | 82 | 76.43 |
| *Diatoma mesodon (Ehrenberg) Kützing* | DMES |  |  |  |  |  |  | 1.45 | 0.75 | 1.49 |
| *Diatoma moniliformis Kützing* | DMON |  |  |  |  |  |  | 0.48 |  | 0.5 |
| *Encyonema ventricosum (Kützing) Grunow in Schmidt & al. var. ventricosum* | ENVE |  |  |  |  |  |  | 2.41 |  | 0.5 |
| *Fragilaria recapitellata Lange-Bertalot & Metzeltin* | FRCP |  |  |  |  |  |  | 2.89 | 1.25 | 1.99 |
| *Gomphonema micropus Kützing var. micropus* | GMIC |  |  |  |  |  |  | 0.48 |  |  |
| *Gomphonema olivaceum (Hornemann) Brébisson var. olivaceum* | GOLI |  |  |  |  |  |  | 1.45 | 0.75 | 1.74 |
| *Gomphonema parvulum var. exilissimum Grunow in Van Heurck* | GPXS |  |  |  |  |  |  | 0.48 |  | 0.25 |
| *Meridion circulare var. circulare (Greville) C.A.Agardh* | MCIR |  |  |  |  |  |  | 0.48 | 0.25 |  |
| *Nitzschia recta Hantzsch in Rabenhorst var. recta* | NREC |  |  |  |  |  |  | 0.24 |  |  |
| *Cocconeis placentula var. euglypta (Ehrenberg) Grunow* | CPLE |  |  |  |  |  |  |  | 0.25 |  |
| *Fragilaria arcus var. arcus (Ehrenberg) Cleve* | FARC |  |  |  |  |  |  |  | 0.25 |  |
| *Gyrosigma sciotoense (Sullivan et Wormley) Cleve* | GSCI |  |  |  |  |  |  |  | 0.25 |  |
| *Caloneis bacillum (Grunow) Cleve var. bacillum* | CBAC |  |  |  |  |  |  |  |  | 0.5 |

## **Table S2.** Summary of methods used by each participant for the experiments E3. The information was gathered based on the description from the providers documentation.

| **Participant** | **RL** | **F** | **D** | **P** | **O** | **I** | **J** | **K** | **B** | **N** |
| --- | --- | --- | --- | --- | --- | --- | --- | --- | --- | --- |
| Brand | Macherey Nagel | Home-made | Macherey Nagel | Qiagen | Qiagen | Qiagen | Qiagen | Qiagen | Qiagen | Macherey Nagel |
| Matrix | Soil | Multi | Plant | Biofilm | Soil | Soil | Plant | Plant | Soil | Soil |
| Lysis device | horizontal | no | no | vertical | horizontal | horizontal | no | no | horizontal | horizontal |
| Mechanical lysis | beads | no | no | beads | beads | beads | no | no | beads | beads |
| Chemical lysis | yes | yes | yes | yes | yes | yes | yes | yes | yes | yes |
| Lysis temperature | no | yes | yes | no | yes | no | yes | yes | no | no |
| Enzymatical Lysis | no | yes | yes | no | yes | no | yes | yes | no | no |
| ProteinaseK | no | yes | no | no | yes | no | yes | no | no | no |
| Rnase_A | no | no | yes | no | no | no | yes | yes | no | no |
| Lysosyme | no | yes | no | no | no | no | no | no | no | no |
| Purification with column | yes | no | yes | no | no | no | no | no | no | yes |
| Purification with centrifugation | no | no | no | yes | yes | yes | no | no | yes | no |
| Precipitation with column | yes | no | yes | yes | yes | yes | yes | yes | yes | yes |

## **Table S3.** Summary of methods used by each participant for the experiments E4. The information was gathered based on the description from the providers documentation

| **Participant** | **RL** | **B** | **E** | **F** | **I** | **J** | **K** | **N** | **O** | **P** |
| --- | --- | --- | --- | --- | --- | --- | --- | --- | --- | --- |
| Brand | Takara | Qiagen | Invitrogen | Takara | KAPA | Thermo-fisher | Qiagen | Thermo-Scientific | Invitrogen | Roche |
| DNA-proofreading | yes | no | no | yes | no | no | no | no | no | no |
| long-range | yes | no | no | yes | no | yes | no | no | no | no |
| 3’-5’ exonuclease activity | yes | no | no | yes | no | no | no | no | no | no |
| “High fidelity” | yes | no | no | yes | no | no | no | yes | no | no |
| “High efficiency” | no | no | no | yes | no | no | no | no | no | no |
| “High sensitivity” | no | no | no | yes | no | yes | no | no | yes | yes |
| Hot start | no | no | no | no | yes | no | no | no | yes | yes |
| 5’-3’ exonuclease activity | no | yes | yes | no | yes | no | yes | no | yes | no |
| Extra A addition | yes | yes | no | yes | no | no | yes | no | yes | yes |
| dUTP incorporation | no | no | no | no | no | no | no | no | no | yes |
| 3A overhang | no | no | yes | no | no | yes | no | no | no | no |
| GC rich template | no | yes | no | no | yes | no | yes | yes | no | yes |

## **Table S4.** Results of the PERMANOVA test conducted with the “participant” factor for the E3 experiment (comparison of DNA extraction methods) with the lake, river and mock samples. D. f.; F; R2 and p correspond to degrees of freedom; F ratio; coefficient of determination and p-value, respectively.

|  | **Factor** | **D.f.** | **Sums of squares** | **Mean of squares** | **F** | **R^2^** | ***p*** |
| --- | --- | --- | --- | --- | --- | --- | --- |
| Lake | Participant | 9 | 0.4415 | 0.049055 | 27.869 | 0.92615 | 0.000999 |
|  | Residuals | 20 | 0.0352 | 0.001760 | 0.07385 |  |  |
|  | Total | 29 | 0.4767 | 1.00000 |  |  |  |
| River | Participant | 9 | 0.124602 | 0.0138447 | 20.86 | 0.90373 | 0.000999 |
|  | Residuals | 20 | 0.013274 | 0.0006637 | 0.09627 |  |  |
|  | Total | 29 | 0.137876 | 1.00000 |  |  |  |
| Mock | Participant | 9 | 0.58346 | 0.064829 | 34.225 | 0.93903 | 0.000999 |
|  | Residuals | 20 | 0.03788 | 0.001894 | 0.06097 |  |  |
|  | Total | 29 | 0.62134 | 1.00000 |  |  |  |

## **Table S5.** Results of the PERMANOVA test conducted with the “Participant” factor for the E4 experiment (comparison of PCR1 methods) with the lake, river and mock samples.

|  | **Factor** | **D.f.** | **Sums of squares** | **Mean of squares** | **F** | **R^2^** | ***p*** |
| --- | --- | --- | --- | --- | --- | --- | --- |
| Lake | Participant | 9 | 0.087353 | 0.0097059 | 17.022 | 0.88966 | 0.000999 |
|  | Residuals | 19 | 0.010834 | 0.0005702 | 0.11034 |  |  |
|  | Total | 28 | 0.098187 | 1.00000 |  |  |  |
| River | Participant | 9 | 0.077682 | 0.0086314 | 17.226 | 0.88573 | 0.000999 |
|  | Residuals | 20 | 0.010022 | 0.0005011 |  | 0.11427 |  |
|  | Total | 29 | 0.087704 |  |  | 1.00000 |  |
| Mock | Participant | 9 | 0.054738 | 0.006082 | 23.396 | 0.91326 | 0.000999 |
|  | Residuals | 20 | 0.005199 | 0.000260 |  | 0.08674 |  |
|  | Total | 29 | 0.059937 |  |  | 1.00000 |  |

## **Table S6.** Results of the PERMANOVA test conducted with the “Enzymatical lysis” factor for the E3 experiment (comparison of DNA extraction methods) with the lake, river and mock samples.

|  | **Factor** | **D.f.** | **Sums of squares** | **Mean of squares** | **F** | **R^2^** | ***p*** |
| --- | --- | --- | --- | --- | --- | --- | --- |
| Lake | Enzymatical lysis | 4 | 0.34444 | 0.086111 | 16.277 | 0.72256 | 0.000999 |
|  | Residuals | 25 | 0.13226 | 0.005290 | 0.27744 |  |  |
|  | Total | 29 | 0.47670 | 1.00000 |  |  |  |
| River | Enzymatical lysis | 4 | 0.091091 | 0.0227728 | 12.169 | 0.66067 | 0.000999 |
|  | Residuals | 25 | 0.046785 | 0.0018714 | 0.33933 |  |  |
|  | Total | 29 | 0.137876 | 1.00000 |  |  |  |
| Mock | Enzymatical lysis | 4 | 0.49685 | 0.12421 | 24.943 | 0.79963 | 0.000999 |
|  | Residuals | 25 | 0.12450 | 0.00498 | 0.20037 |  |  |
|  | Total | 29 | 0.62134 | 1.00000 |  |  |  |

# Supplementary figures

## **Figure S1.** Instructions given to the participants for the experiment E1


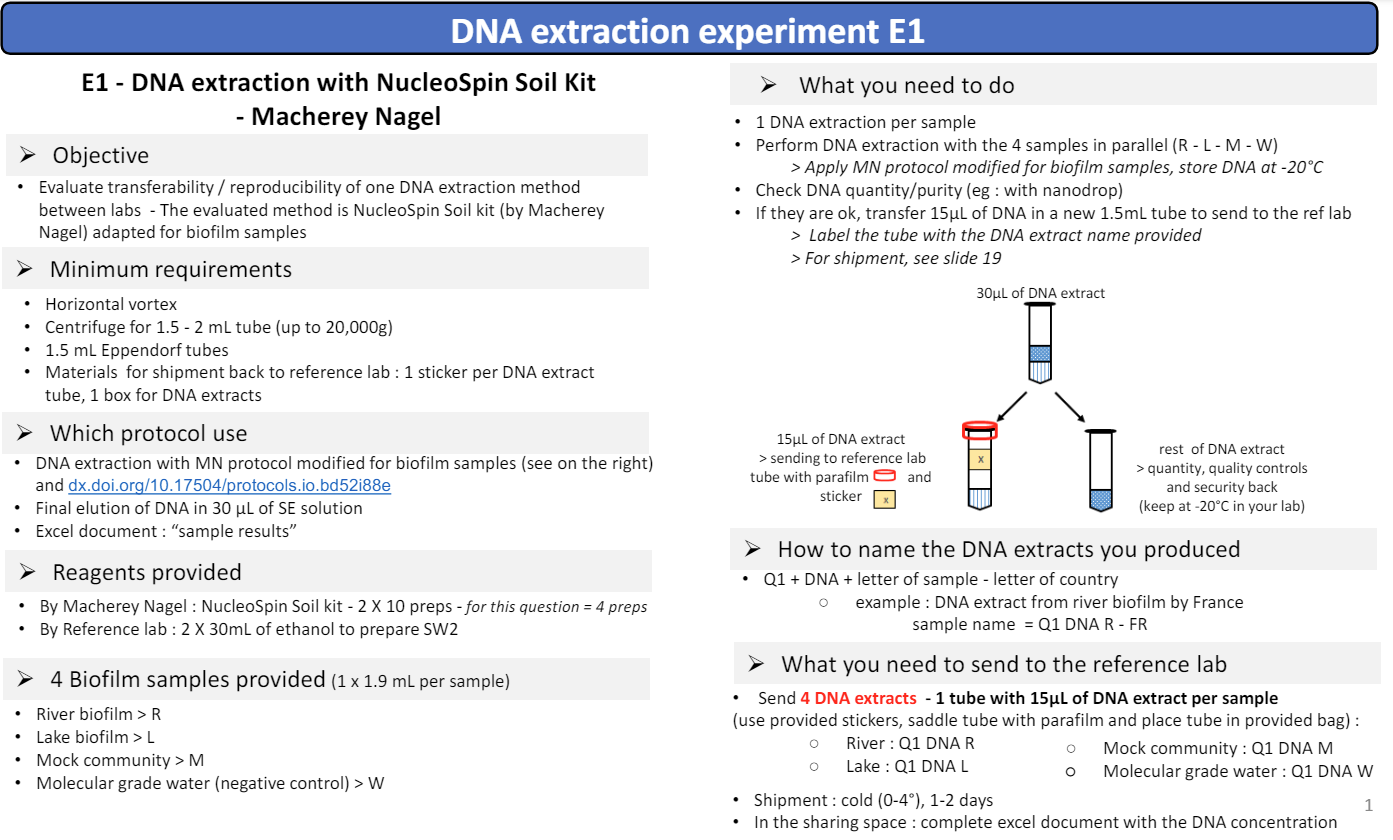


## **Figure S2.** Instructions given to the participants for the experiment E2


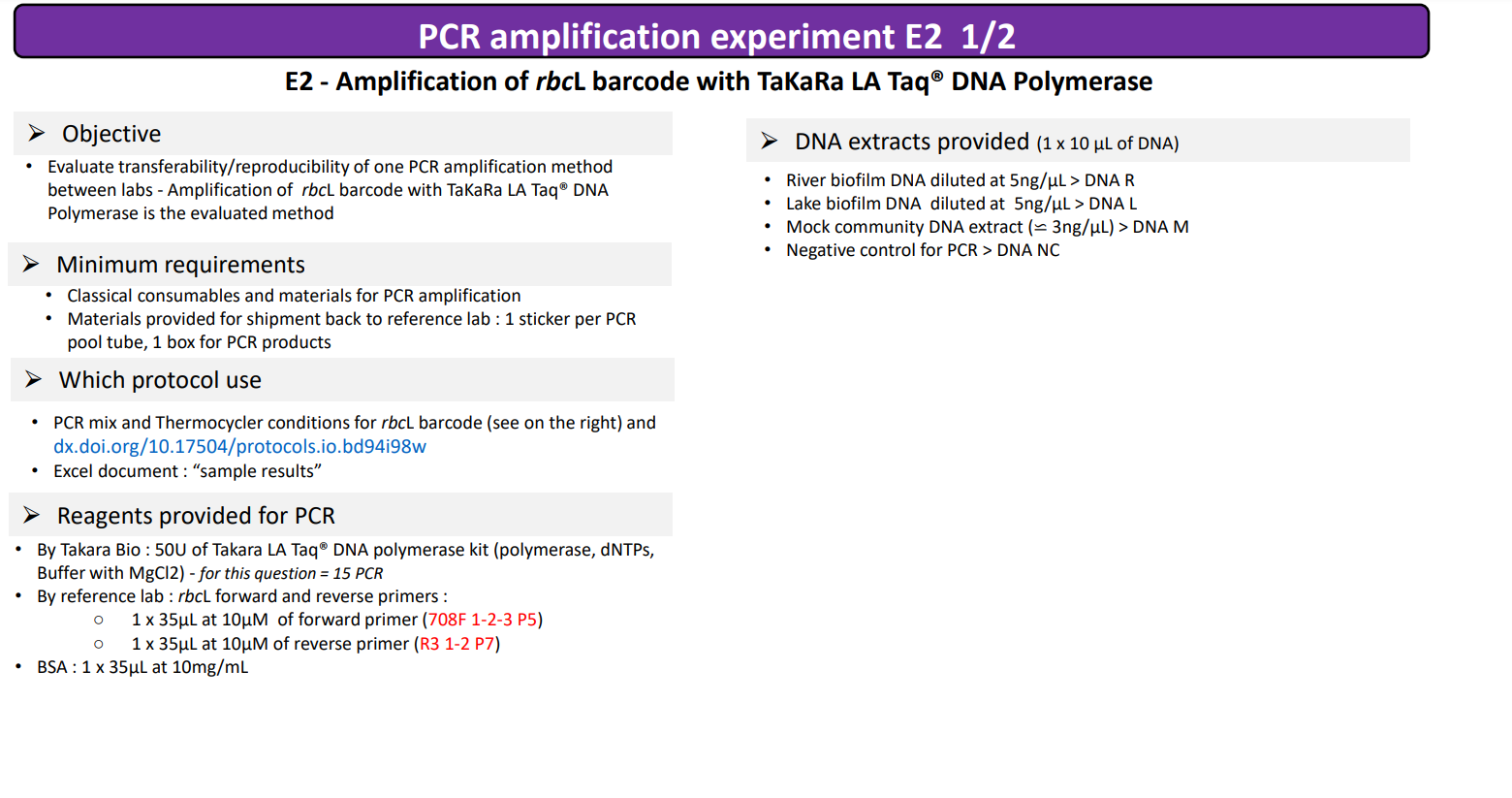


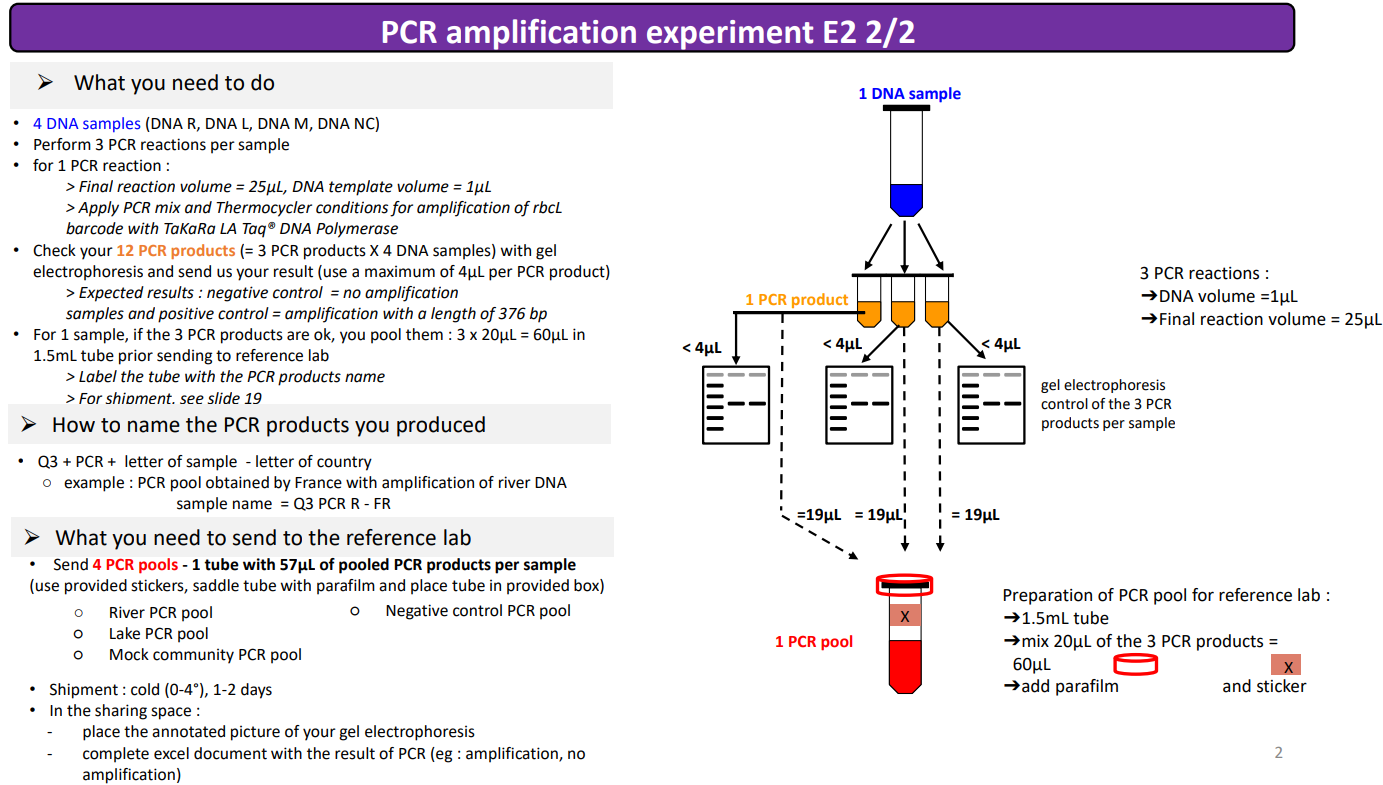


## **Figure S3.** Instructions given to the participants for the experiment E3


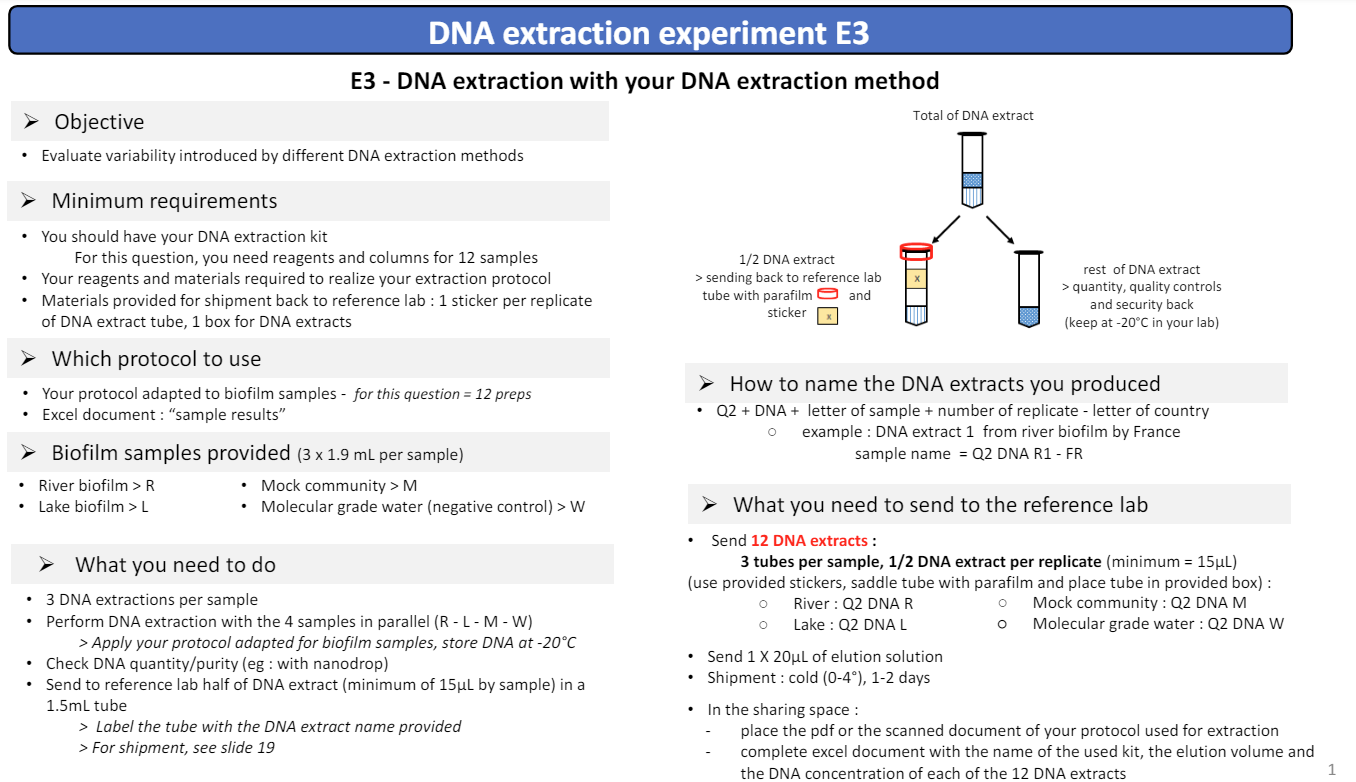


## **Figure S4.** Instructions given to the participants for the experiment E4


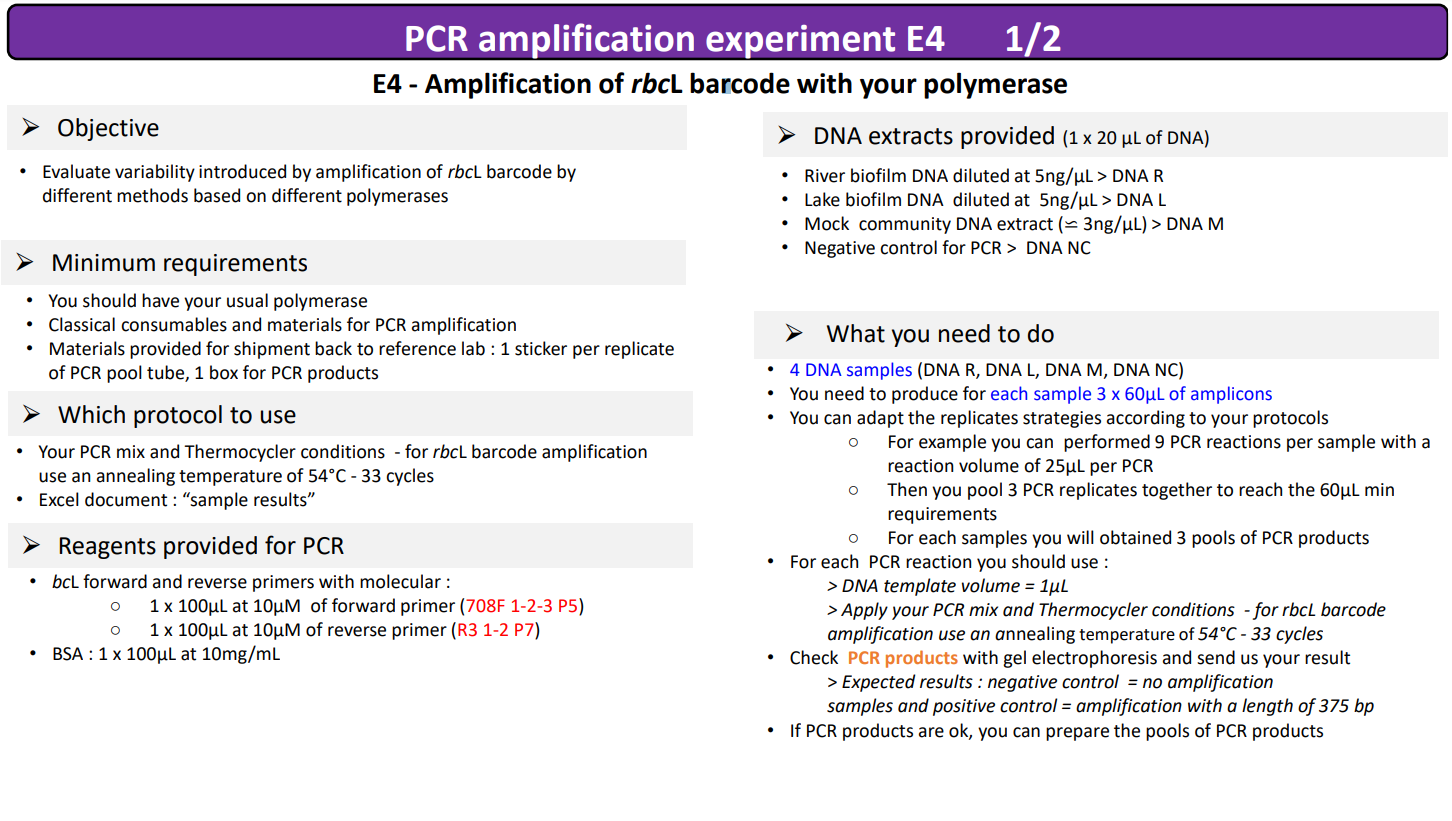


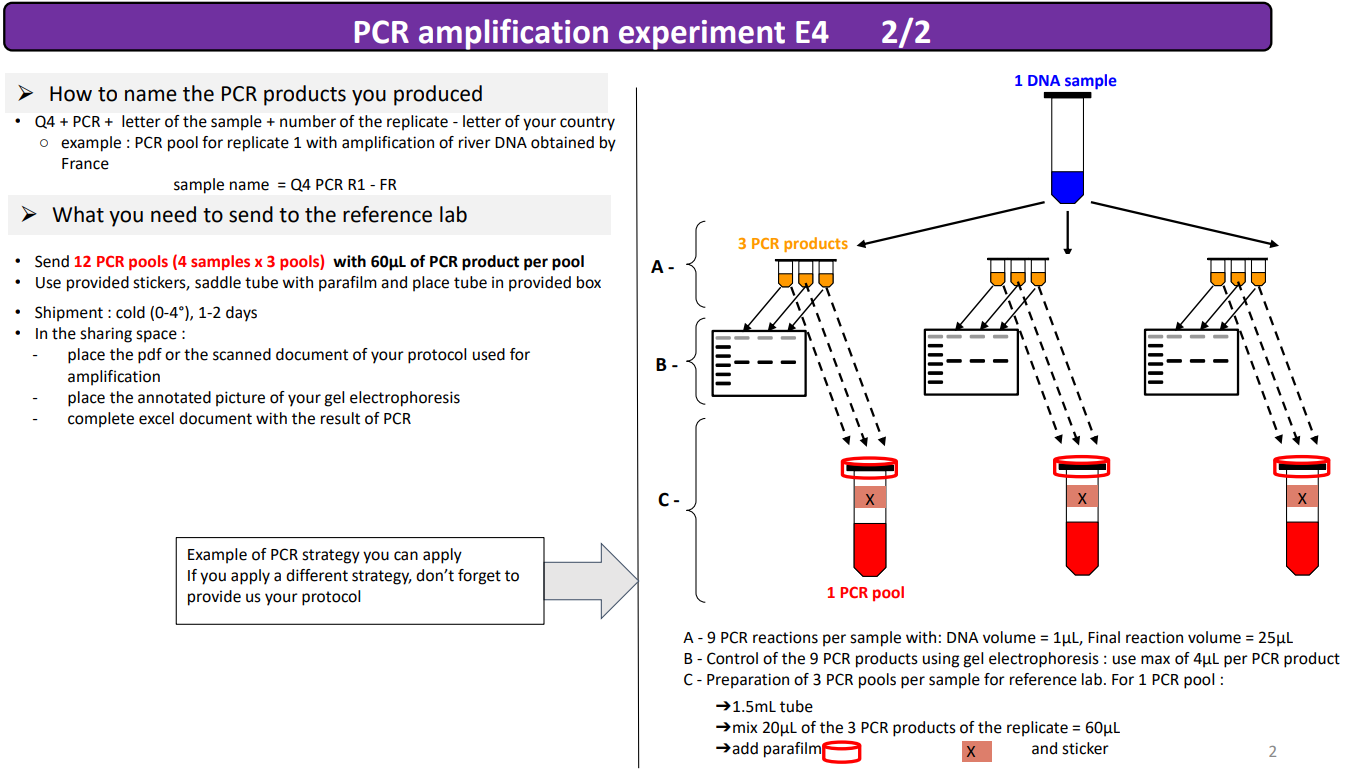


## **Figure S5.** Community composition and NMDS plots performed with the Bray-Curtis dissimilarity index calculated with the datasets obtained from E3 experiment, with the mock, river and lake samples. Left side NMDS plots display the participants codes. Right side NMDS plots display the different extractions methods according to the use of proteinase K, Rnase A and Lysosime.


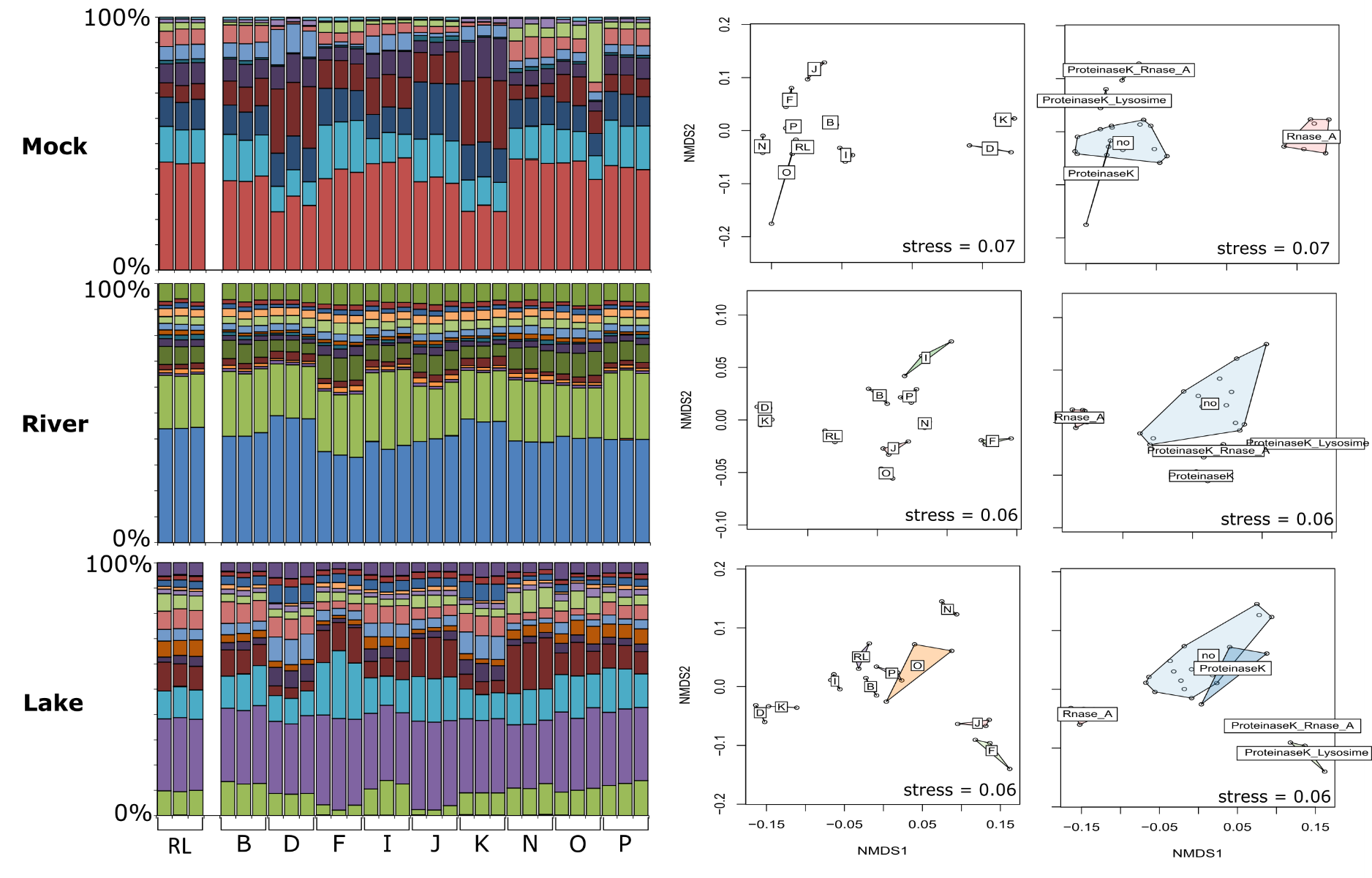

Supplement: Supplement1 [file NIHMS2054293-supplement-Supplement1.docx]
